# Artificial intelligence in clinical thrombosis and hemostasis: A review

**DOI:** 10.1016/j.rpth.2025.102984

**Published:** 2025-07-24

**Authors:** Yi Kiat Isaac Kuan, Yixin Jamie Kok, Nigel Sheng Hui Liu, Brandon Jin An Ong, Ying Jie Chee, Chuanhui Xu, Minyang Chow, Kollengode Ramanathan, Rinkoo Dalan, Prahlad Ho, Bingwen Eugene Fan

**Affiliations:** 1 Department of Haematology, Tan Tock Seng Hospital, Singapore; 2 Yong Loo Lin School of Medicine, National University of Singapore, Singapore; 3 Lee Kong Chian School of Medicine, Nanyang Technological University, Singapore; 4 Department of Endocrinology, Tan Tock Seng Hospital, Singapore; 5 Department of Rheumatology, Allergy and Immunology, Tan Tock Seng Hospital, Singapore; 6 Department of General Medicine, Tan Tock Seng Hospital, Singapore; 7 Cardiothoracic Intensive Care Unit, National University Heart Centre, National University Health System, Singapore; 8 Department of Haematology, Northern Hospital, Epping, Victoria, Australia; 9 Northern Clinical Diagnostics and Thrombovascular Research (NECTAR) Centre, Northern Health, Epping, Victoria, Australia; 10 Department of Medicine (Northern Health), University of Melbourne, Epping, Victoria, Australia; 11 Department of Laboratory Medicine, Khoo Teck Puat Hospital, Singapore; 12 Department of Laboratory Medicine, Woodlands Health Campus, Singapore

**Keywords:** artificial intelligence, machine learning, thrombosis, hemostasis, hemorrhage

## Abstract

**Background:**

Artificial Intelligence (AI) and machine learning (ML) are transforming hemostasis and thrombosis care, with applications spanning disease detection, risk assessment, laboratory testing, patient education, personalized medicine, and drug development. This narrative review explores AI’s clinical utility and limitations across these 6 domains.

**Methods:**

A comprehensive search of PubMed, Embase, and Scopus (up to February 2025) was conducted using terms related to AI, thrombosis, and hemostasis. Peer-reviewed, English-language studies were included, supplemented by manual and reference screening. Of 84 studies included, 38 focused on risk assessment, 16 on diagnostics, and others on personalized medicine, drug development, and patient engagement.

**Results:**

AI demonstrated high accuracy in diagnosing thrombotic events via imaging and electronic health record analysis, although sensitivity gaps persisted for complex cases. In laboratory settings, AI outperformed manual review in detecting errors (eg, sample mislabeling and clotted specimens). Risk stratification models surpassed traditional scores (eg, CHA_2_DS_2_-VASc) in predicting thromboembolism, yet inconsistently performed in cancer-associated thrombosis. Personalized anticoagulation dosing and genetic severity prediction in hemophilia highlighted AI’s precision. Chatbots and adherence tools have enhanced patient education while AI-driven drug discovery identified novel anticoagulants and repurposed existing therapies. Limitations included variable external validation, “black box” interpretability issues, and dataset biases.

**Conclusion:**

AI offers significant promise for improving diagnostics, risk prediction, and individualized therapy in thrombosis and haemostasis. Future integration depends on transparent, validated, and equitable AI systems embedded within clinical workflows.

## Essentials

• We reviewed AI studies (2017–2025) across radiology, laboratory, and drug development settings.
• AI enhances care in thrombosis and hemostasis via data-driven diagnosis and risk prediction.
• AI tools improve diagnostics and improves risk scores for thrombotic and bleeding risk.
• Challenges include interpretability and validation, requiring governance for safe adoption.

## 1 Introduction

Artificial intelligence (AI) involves creating systems that replicate human-like cognitive abilities, enabling machines to perform complex functions such as learning, reasoning, problem solving, and language processing. These systems are driven by advanced algorithms and large datasets, allowing them to autonomously refine their performance and adapt through experience. Within AI, machine learning (ML) serves as a key subfield that allows computers to identify patterns and make predictions from complex datasets without explicit programming. ML algorithms—ranging from logistic regression and decision trees to more advanced deep learning techniques—are increasingly leveraged to analyze vast amounts of clinical data, improving efficiency and accuracy in patient care. In the context of hemostasis and thrombosis, the applications of AI-driven technologies span far and wide, from research and pathophysiology, through laboratory diagnostics and the identification and validation of disease biomarkers, to clinical diagnostics and prediction tools. These advancements rely on AI’s ability to synthesize and interpret multimodal data, including laboratory test results, electronic health records (EHRs), imaging studies, and genetic profiles.

This narrative review aimed to explore these aspects in detail, focusing on 6 key areas: disease detection and diagnosis, risk assessment and stratification, streamlining laboratory testing, improving patient education, personalized medicine, and accelerating drug development; providing insights into AI’s transformative role in thrombosis care and the hurdles that must be addressed for its successful integration into routine clinical workflows.

### 1.1 Concepts in AI and ML

**Figure 1:**
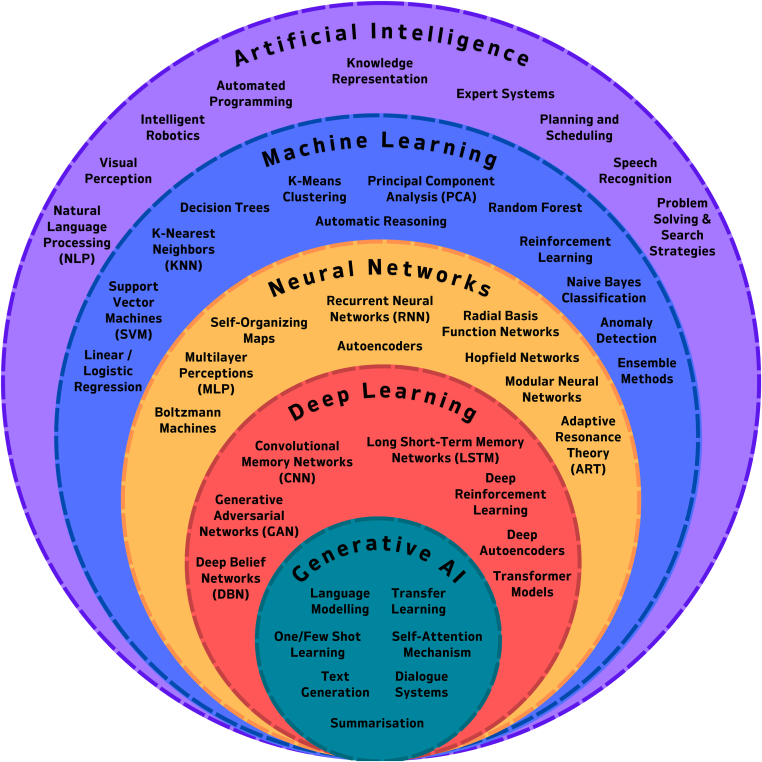
Artificial intelligence (AI) and its subsets.

AI refers to the simulation of human intelligence in machines, enabling them to perform tasks that require cognitive abilities such as problem solving, decision making, learning, and adaptation (Figure 1). Key subfields of AI include natural language processing (NLP) and ML. ML allows computers to identify patterns and make predictions from data without explicit programming. It can be trained to process various types of data, including numerical values, time series, and images. A subset of ML, deep learning, focuses on artificial neural networks (ANNs). These networks consist of layers of interconnected nodes, where each layer passes weighted information to the next. The model adjusts these weights to produce an output based on the input [1], making deep learning particularly effective for processing complex data sets. Unlike traditional ML models, ANNs are often referred to as black boxes because they handle massive arrays of parameters (up to millions or even billions) and their internal decision-making processes (determined by the relative weights of each parameter within the intervening or “hidden” layers) are difficult to interpret. A further subset of deep learning is generative AI, where generative models are used to produce new data such as text, images, or videos by learning the underlying patterns of their training data.

Another important area of AI that intersects with deep learning and generative AI is NLP. NLP enables machines to process text and speech data, generating human-like language. Regardless of the model type, ML systems must be trained on data, which can be either labeled or unlabeled. Models trained on labeled data are part of supervised learning, where the goal is to replicate patterns labeled by humans [2]. In contrast, unsupervised learning models are trained on unlabeled data, seeking to identify inherent patterns without prior labels.

Training ML models typically involves splitting data into subsets: a training set, a validation set, and a test set. The training set exposes the model to patterns and relationships, the validation set fine-tunes the model’s parameters, and the test set evaluates its generalizability on unseen data. A key challenge in ML is avoiding overfitting, where a model becomes too tailored to the training data and fails to generalize to new cases. Conversely, underfitting occurs when a model is too simplistic and cannot capture meaningful relationships in the data, thus lacking predictive value [3]. Proper validation, high quality data and external testing are essential for addressing these challenges, ensuring the model performs well in real-world situations.

## 2 Methods

We conducted a search to identify all potentially relevant publications about artificial learning-based applications in thrombosis research, diagnosis, and clinical management. A narrative review of the literature is presented using various databases such as PubMed, Embase, and Scopus for all articles indexed up to February 2025. Our search strategy (Supplementary Material) involved Medical Subject Heading terms and free-text keywords involving all 3 of the following domains: (1) “Artificial Intelligence,” “Machine Learning,” “Neural Networks.” (2) “Thrombosis.” (3) “Haemostasis,” “Bleeding.” Following this, we also included a manual search strategy with searches in google scholar along with the abovementioned databases with targeted search terms in particular fields of interest such as “disease diagnostics,” “personalized medicine,” “patient education,” “laboratory testing,” “risk assessment,” and “drug development.” We also reviewed the references of included articles to identify further articles of interest. Screening of articles and data extraction were conducted by 5 independent reviewers (Y.K.I.K., Y.J.K., N.S.H.L., B.J.A.O., and B.E.F.). Disagreements were solved by discussion, during which 2 other reviewers were involved (B.E.F. and Y.K.I.K.).

**Figure 2:**
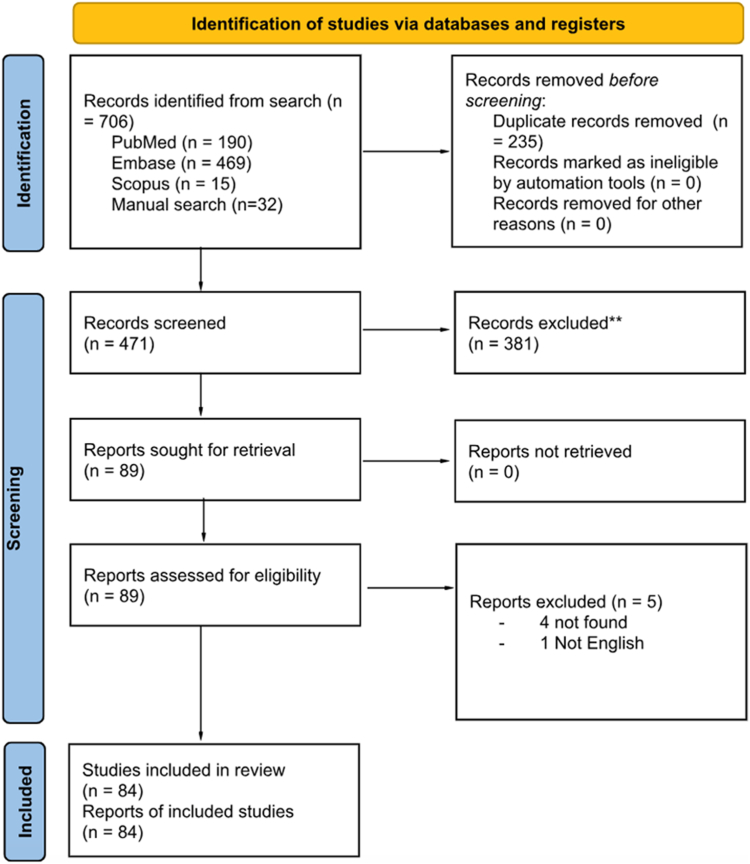
Prisma flow diagram of literature review.

**Figure 3:**
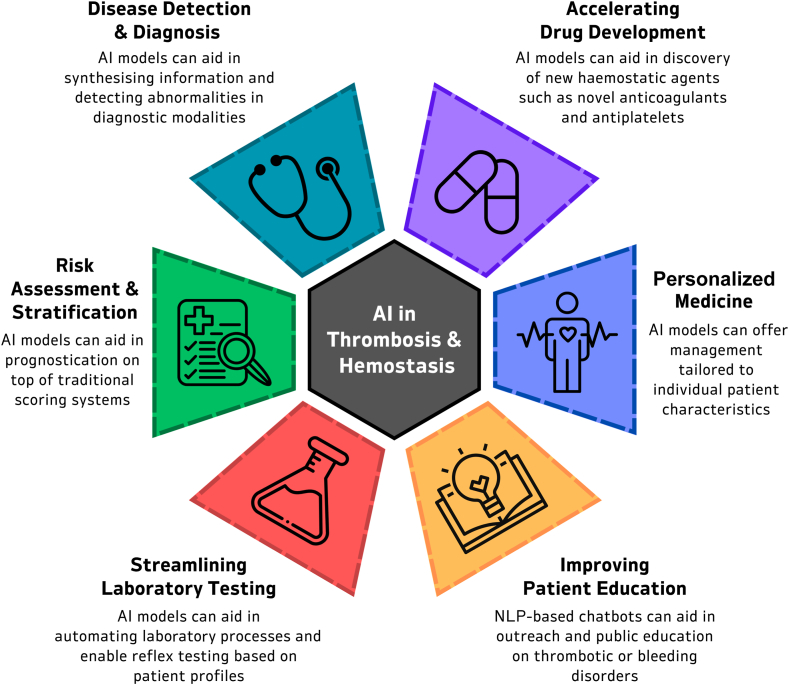
Applications of artificial intelligence (AI) in thrombosis and hemostasis.

The main requirement for inclusion was the description and evaluation of AI-based methods or tools yielding applications in thrombosis and hemostasis research. To be included in this review, the key terms had to be identified in either the title or abstract of a full-text article. Only full, peer-reviewed articles in English were included. We incorporated pertinent large studies and others with relevant findings in this narrative review. A total of 84 references met the inclusion criteria (Figure 2); 14 studies were included that were relevant to AI and ML in disease diagnostics, 16 were relevant to personalized medicine, 2 were relevant to patient education and medication compliance, 5 were relevant to laboratory testing in thrombosis and hemostasis, 38 were relevant to risk assessment and risk stratification, 9 were relevant to accelerating drug development (Figure 3).

### 3 Disease Detection and Diagnosis

AI and ML models have increasingly been applied to improve the detection, diagnosis, and management of various bleeding and thrombotic disorders. These models have been applied to both imaging modalities and to clinical tests and diagnostic criteria.

### 3.1 AI in imaging and text modalities

AI can detect clots from various imaging modalities, including iliofemoral deep venous thrombosis (DVT) from computed tomography (CT) angiography [4], DVT from ultrasound [5], and incidental pulmonary embolism (PE) from contrast–enhanced chest CT with sample sizes ranging from >200 to >2000 patients [6]. Kainz et al. [5] explored the use of a deep learning model for the automatic interpretation of compression ultrasound images in suspected DVT cases. By integrating AI into ultrasound diagnostics, they demonstrated DVT examination by nonspecialists in a primary care setting is feasible, enabling more accessible point-of-care DVT diagnosis [5]. These radiology applications also extend to bleeding disorders. Nagao et al. [7] used AI-assisted ultrasound imaging with an AI algorithm trained on 3435 images from multiple centers to detect hemarthrosis and synovitis in people with hemophilia, achieving an area under the curve (AUC) of at least 0.87 [7]. Another AI model trained on 142 patients was able to detect active bleeding in mesenteric and celiac artery angiography to an accuracy of 77.3% [8].

In real-world settings, the Brainomix e-Stroke software is used to assist in interpreting CT imaging for time-sensitive acute stroke detection. Mallon et al. [9] studied the performance of the software on 551 stroke unit patients and found the software was highly accurate (sensitivity, 100%; specificity, 97.6%) in identifying hemorrhages and also had a 77% accuracy in automated interpretation of non-contrast CT of the head scoring to assess if a patient would benefit from mechanical thrombectomy, with only 0.6% of patients misclassified as ineligible [9]. Nonetheless, limitations do exist: the software had a lower sensitivity of only 65% for identifying thrombotic occlusions, with significant false-negative results especially for medium vessel occlusions. Moreover, Batra et al. [6] reported that while their incidental PE detection model had a high negative predictive value (99.8%), its positive predictive value was lower than that of radiologists. In real-world practice, low positive predictive value and low sensitivity in AI tools can undermine their effectiveness. As seen in the PE model by Batra et al. [6], frequent false-positive results can lead to unnecessary tests, increased workload, and reduced clinician trust. In contrast, the Brainomix e-Stroke software’s low sensitivity for thrombotic occlusions risks missing treatable strokes, potentially delaying critical interventions like thrombectomy. These limitations highlight the need for AI to support—not replace—clinical judgment, especially in time-sensitive or high-stakes scenarios.

Beyond imaging, NLP AI models have been used to analyze EHRs and extract significant findings such as identifying venous thromboembolism (VTE) from radiology reports [[10], [11], [12]]. NLP interrogation of radiology reports from ultrasound, CT pulmonary angiography, CT angiography of the chest, and ventilation/perfusion scans yielded high sensitivity (99.6%) and specificity (93.3%) in identifying VTE [10] in a California–based study based on 400 patient encounters. Similarly, Pedersen et al. [13] developed a deep learning model to detect bleeding events in unstructured EHR text (sensitivity 90%, specificity 90%). For clinical translation, such NLP models that extract VTE or bleeding events from unstructured EHR data may enable automated, high-accuracy construction of epidemiological databases. These can support local and multicenter registries, enhance disease surveillance, and facilitate large-scale research without relying on delayed or incomplete coding. Such tools also allow for real-time risk stratification and outcome tracking to inform clinical decision making and public health planning.

### 3.2 AI applied in clinical testing and diagnostic criteria

AI tools are useful aids in conventionally challenging and complex diagnoses. In a multicenter prospective observational study on 1393 patients from 10 centers, Nilius et al. [14] developed an AI model to improve heparin-induced thrombocytopenia diagnosis, significantly reducing the number of false-negative and false-positive cases when compared with traditional diagnostic algorithms such as the 4Ts score and immunoassays. False-negative results were reduced by at least 45% when compared with 3 immunoassays, while false-positive results reduced by 53% to 72% for 2 immunoassays but increased by 29% for 1 immunoassay [14]. AI has also been leveraged in disseminated intravascular coagulation (DIC) diagnosis, with an externally validated AI model by Yoon et al. [15] outperforming conventional scoring systems with an AUC of 0.96 to 0.98 [15]. Another application has been to more consistently and conveniently diagnose antiphospholipid syndrome (APS). Current APS diagnostic criteria requires both identification of specific autoantibodies and meeting clinical criteria, which can be prone to interassay and interlaboratory variation and require interruption of anticoagulation. de Laat-Kremers et al [16] explored the application of neural network models to use thrombin generation data to diagnose APS, achieving an accuracy up to 91%, and repeated similar results even in anticoagulated patients [17]. While these findings suggest that AI could overcome the limitations of standard laboratory assays, the study’s relatively small sample size (81 patients with APS, 126 control patients in total in both training and validation cohorts) and lack of external validation limit generalizability.

Together, these applications highlight AI’s potential to improve diagnostic precision and streamline clinical decision making in thrombosis and hemostasis. Furthermore, it can allow surveillance of an entire health care organization’s population beyond just subsamples [11].

### 3.3 Streamlining laboratory testing

AI is increasingly being integrated into laboratory medicine to enhance the efficiency, accuracy, and reliability of testing. A critical focus is the preanalytical phase, which is particularly prone to human error. Issues such as sample mislabeling, clot contamination, and sample mix-up can significantly compromise diagnostic accuracy and patient safety. To address these challenges, AI models have shown strong performance in detecting preanalytical errors.

Zhou et al. [18] introduced a deep learning-based delta check system using a Deep Belief Network to detect sample mix-ups by analyzing 22 routine hematology test items, with 423,290 test results. This model achieved a test accuracy of 93.1%, significantly outperforming traditional statistical delta check methods, which had lower accuracy and higher false-positive rates [18]. Fang et al. [19] used backpropagation neural networks trained on 3081 samples (of which 192 were clotted) to automatically detect clotted blood specimens in coagulation testing, classifying samples to 97% accuracy and therefore reducing the risk of reporting erroneous results [19]. Lippi et al [20] discussed a broader vision for AI in pre-analytical automation, including automated patient identification and labeling, robotic phlebotomy, and intelligent specimen routing, all of which minimize variability in sample processing and reduce turnaround time.

AI applications extend beyond error detection, offering enhanced interpretation and application of laboratory coagulation assays. Arumugam et al. [21] used random forest models to analyse thrombin generation kinetics and distinguish acute coronary syndrome from stable coronary artery disease. Their approach used high-dimensional clotting cascade simulations and identified novel coagulation features that distinguished hypercoagulability in acute coronary syndrome to 87.2% accuracy [21]. In another study, Qian et al. [22] developed Coagulo-Net, a physics-informed neural network that integrates mechanistic models of coagulation with deep learning to enable inference of unknown reaction constants and improve prediction accuracy.

### 3.4 Risk assessment and stratification

AI can be integrated into risk assessment and stratification for thrombotic and bleeding disorders. Traditional clinical risk scores, such as CHA_2_DS_2_-VASc and HAS-BLED, rely on demographic and laboratory parameters but have limitations in predictive accuracy. AI models can incorporate a broader range of variables and train on more extensive, diverse datasets, allowing for improved predictive accuracy.

AI models have been successfully used to predict postprocedural complications such as VTE and major bleeding, for a wide variety of procedures including cardiac surgery [23], extracorporeal membrane oxygenation [24], bariatric surgery [25], total joint arthroplasty [26], radical gastrectomy [27], inferior vena cava filter placement [28], and catheter ablation [29]. AI models applied to large datasets across these different clinical settings have shown high predictive performance (AUCs ranging from ∼0.75-0.97), particularly when postoperative variables were incorporated. Strengths include the use of interpretable models (eg, permutation feature importance, LASSO, and decision trees), granular data, large sample sizes in some studies (with the largest comprising 666,772 patients across 1080 centers) [23] and validation cohorts, supporting their potential for personalized risk stratification. However, limitations remain; most models lack prospective validation and may be subject to bias from retrospective design or outcome-related variables that risk reverse causation (eg, transfusions predicting bleeding requiring reoperation). AI has also shown utility in the management of anticoagulation therapy in atrial fibrillation (AF) by predicting risk of stroke, major bleeding, and all-cause mortality, sometimes outperforming traditional risk scores like CHA_2_DS_2_-VASc for stroke and HAS-BLED for bleeding [30,31]. Goto et al. [32] also demonstrated that an AI model trained on serial international normalized ratio values during warfarin initiation could predict outcomes up to 1 year into treatment, with better accuracy than time-in-therapeutic-range metrics, which is the current gold standard [32]. However, 1 AI model trained on 18,388 patients with AF and cancer had poor bleeding risk prediction (despite also achieving high accuracy in stroke prediction), underscoring the complexity of bleeding predictions [33].

Several studies highlight AI’s role in improving VTE risk stratification. A meta-analysis of 12 studies evaluating AI’s performance in VTE prediction found consistent sensitivity and specificity across thrombus type and type of AI method used, although 7 of the 12 studies only had a training set and lacked a test set [34]. A randomized trial of 19,785 hospitalized patients showed AI-based systems reduced VTE incidence by 46% and increased mechanical prophylaxis by 24% [35]. However, inappropriate prophylaxis was sometimes applied to high-bleeding risk patients and overall prophylaxis rates did not change. Other studies demonstrated AI models achieving high accuracy in predicting DVT [36], and PE mortality and recurrence risk after premature anticoagulation discontinuation [37]. When compared with traditional risk scores, AI models outperformed the IMPROVE score in VTE prediction [38] and stratified risk better than the Caprini Risk Score [39]. However, for PE-related mortality prediction, AI models only marginally outperformed simplified pulmonary embolism severity index and pulmonary embolism severity index scores [40]. AI has shown promising results when applied to specific prothrombotic conditions, including achieving 98% to 100% positive predictive value for thrombosis in COVID-19 patients [[41], [42], [43]], outperforming the Khorana score in predicting VTE in gyneoncology patients [44], and identifying risk of portosplenomesenteric venous thrombosis in acute pancreatitis [45].

AI has also advanced bleeding risk assessment, particularly in anticoagulated patients. A study of AI models on 300,000 anticoagulated patients found superior performance to HAS-BLED in predicting gastrointestinal bleeding [46], while another study showed they outperformed the IMPROVE model in predicting major bleeding [47]. However, a prospective cohort study by Fard et al. [48] with 2542 patients showed their AI models performed only comparably with traditional scores [48]. Interestingly, a later study by the same authors found incorporating time-series data improved performance of AI models, demonstrating AI’s potential in dynamic risk assessment [49]. AI tools have also been used to classify bleeding events and to dynamically predict bleeding risk in intensisve care unit (ICU) patients treated with antithrombotic therapy. In a study involving 1938 ICU patients, all AI classifiers were able to classify events within 1 minute, significantly faster than senior clinicians who took 53 minutes, and to a higher degree of accuracy (>0.7 vs 0.6488) [50]. Aside from bleeding in anticoagulated patients, AI has been used to predict critical bleeding in immune thrombocytopenia [51] and esophageal variceal bleeding in cirrhotic patients [52]. In acute gastrointestinal bleeding, AI models performed comparably with the Glasgow–Blatchford score in predicting intervention needs [53]. Similarly, Zanaty et al. [54] developed an AI model predicting subdural hemorrhage recurrence in patients on long-term anticoagulation and identified an optimal window from day 2 to day 20 for anticoagulation resumption.

However, there are some notable exceptions. In cancer-associated thrombosis, AI models outperformed the CAT-BLEED score in terms of discrimination [55,56], but their precision remained low when prioritizing sensitivity—limiting their clinical utility for high-risk patients [55]. In hemodialysis patients, AI approaches demonstrated poor predictive power compared with conventional scores [57]. Some AI models had reduced performance during external validation. For example, Mora et al. [28] developed a bleeding risk model using >49,000 patients from the RIETE registry with strong internal performance. However, when validated externally on the COMMAND-VTE cohort, the model underperformed relative to traditional scores such as RIETE and VTE-BLEED [58]. The drop in performance may be attributed to the absence of 14 baseline variables in the external dataset.

For other coagulation disorders, AI has been applied to predict DIC, outperforming Japanese Association for Acute Medicine DIC and International Society on Thrombosis and Haemostasis (ISTH) sepsis induced coagulopathy criteria [59]. Gradient-weighted class activation mapping has also been used to enhance model interpretability in DIC prediction [60].

### 3.5 Personalized medicine

AI enhances the ability to develop personalized treatment strategies based on patient characteristics, genetic profiles, and real-time clinical data. Unlike traditional 1-size-fits-all approaches, AI models can integrate diverse data sources to optimize dosing, predict risks, and guide thrombosis prevention strategies.

Anticoagulation therapy requires careful dosing to prevent thrombotic events while minimizing the risk of bleeding. Many centers deploy rule-based algorithms and specialist anticoagulation teams to titrate these doses individually based on real-time blood work monitoring. Abdel-Hafez et al. [61] was able to predict activated partial thromboplastin time levels to improve heparin dosing prediction using an AI model trained on data from 3019 patient care episodes from 5 hospitals including external validation. In warfarin therapy, AI models have demonstrated the ability to improve dosing accuracy, performing comparably or better than clinical algorithms or subjective physician decision making [62,63]. Gordon et al. [64] and Petch et al. [6] also showed AI models integrating time-varying data could predict patients at risk of suboptimal anticoagulation control and perform comparably with rule-based dosing algorithms in optimizing warfarin dose adjustments. Gordon et al. [64] trained their models using data from 35,479 patients in a database of linked primary and secondary care data, while Petch et al. [65] used data from 22,502 patients in 3 clinical trials with external validation. AI models, such as decision tree-based approaches, have also been applied to personalize warfarin therapy in elderly patients by predicting drug–drug interactions and optimizing dosing decisions [66]. This individualized approach enhances clinical safety and efficacy, particularly in the context of polypharmacy. An AI model by Huang et al. [67] integrated pharmacogenomic and metabolomic data to predict individual warfarin sensitivity and an effective dose range, demonstrating the utility of AI models to incorporate a wide range of data sources that traditional algorithms may lack. On the opposite spectrum, Gan et al. [68] demonstrated the efficacy of an AI model trained on 118 patients to function in a small data setting to optimize dosages—requiring only warfarin dose and international normalized ratio values as inputs—performing on par with physician-guided dosing [68]. However, caveats do exist, as a systematic review of AI models for personalized heparin dosing found many studies lacked external validation, had small sample sizes, and did not evaluate feasibility in clinical practice [69].

Despite its promise, concerns about AI’s black box nature—where predictions lack clear explanations—remain a challenge. One study addressed this by combining computational modeling with deep learning to simulate thrombin generation and thrombus formation under warfarin, dabigatran, and rivaroxaban therapy, with the resulting ANN achieving 96% accuracy in a virtual patient modeled on the thrombin generation curves of a real patient [70]. This tool uses the thrombin generation assays to make fast predictions of the individual response of patients to anticoagulant therapy, offering a transparent and data-driven approach to personalized anticoagulation management.

AI can also aid in evaluating genetic disorders predisposing to bleeding or other coagulation disorders. In genetic bleeding disorders like hemophilia A (HA), graph-based AI models have been applied to map critical factor (F)VIII mutations and predict disease severity [71,72]. Although HA mostly affects males due to the X-linked nature of the disease, it is now recognized that variants of FVIII in females can result in HA; however, tools to identify at-risk women and girls remain lacking. An AI model trained on male patient data was able to distinguish mild, moderate, and severe cases of HA in female patients, although the limited dataset of only 273 genotyped females may affect generalizability [73]. AI has also improved risk prediction for other genetic disorders by identifying genetic polymorphisms associated with coagulation abnormalities. AI ML studies have identified a susceptibility locus for PE [74], as well as genetic variants that contribute to thrombosis susceptibility [75]. They also identified renin-angiotensin system genetic variants as significant predictors of warfarin-induced bleeding, with patients in the highest risk quartile having a 12-fold increased bleeding risk compared with moderate-risk patients [76].

### 3.6 Improving patient education and compliance

Patient education is a critical component of managing hemostatic and thrombotic disorders, particularly in conditions requiring lifelong anticoagulation or hemophilia care. AI has emerged as a powerful tool to enhance patient adherence, self-management, and education, offering personalized and real-time interventions. However, clinical studies in this sector were limited and often only small scale with <50 participants. Lewandowska et al. [77] explored the integration of AI-driven patient education and self-monitoring technologies in hemophilia care. The study emphasized applications such as patient-performed ultrasonography with AI-assisted scan interpretation and NLPs such as chatbots to support digital educational platforms [77]. In Senegal, a chatbot for persons with hemophilia was developed to address identified disease knowledge gaps. Users found the chatbot informative and easy to use, enabling them to better understand their condition and manage symptoms at home while waiting to see a doctor. The accessibility of a chatbot in their local language was also greatly appreciated, underscoring the role of AI in bridging health care communication barriers and empowering patients in underserved regions.

Moreover, adherence to anticoagulation therapy in stroke patients is crucial, as lapses in medication can significantly increase the risk of stroke or bleeding. Labovitz et al. [78] designed an AI-powered platform that used a smartphone application to visually confirm drug ingestion, providing real-time reminders and adherence tracking. In a 12-week randomized trial with 28 participants, the intervention group, which used the platform, showed a 50% improvement in adherence based on plasma drug concentration levels, compared with the control group who did not receive monitoring. For patients receiving direct oral anticoagulants, absolute improvement increased to 67% [78].

### 3.7 Accelerating drug development

AI has potential in enhancing multiple aspects of drug discovery and treatments for thrombotic disorders. AI models enable the rapid analysis of large datasets, helping to identify new mechanistic insights and potential drug targets, as well as optimizing drug evaluation. AI has been used to analyze and design compounds on a molecular level, streamlining the drug discovery process and allowing for in silico analysis of compounds before moving on to synthesis and in vitro testing.

Xie et al. [79] applied a computational biology algorithm to identify high-risk molecular targets, termed recurrent risk modules (RRMs), which are associated with recurrent VTE [79]. Their novel approach integrated gene expression profiling and human signaling networks and was able to identify RRMs that were key in the pathogenesis of VTE, thereby identifying therapeutic drug targets. Notably, 9 drug targets in the RRMs are already targeted by known anticoagulants, thrombolytics, and antiplatelets. Furthermore, AI-driven molecular modeling is also being applied to the design of anticoagulant and coagulant therapies. Rovenchak et al. [80] used deep learning-based autoencoders to generate novel anticoagulant and coagulant candidates by mapping the chemical space of known inhibitors, after which computational screening further refined these candidates, generating 1571 potential configurations for protein C inhibitors. This is valuable for drug development in conditions with limited experimental data. Similarly, AI has been applied to the rational design of anticoagulants by identifying molecular features influencing thrombin inhibition [81,82]. Masand et al. [81] used an explainable AI model on 2803 thrombin inhibitors, identifying 8 key descriptors with strong internal and external validation. SHapley Additive exPlanations analysis enhanced interpretability, offering insights to support structure-based anticoagulant development [81]. Huang et al. [83] used AI to design and predict dabigatran derivatives, with 3 compounds synthesized and demonstrating bioactivity equivalent to dabigatran.

Beyond designing new drugs, AI has proven valuable in repurposing existing ones and predicting off-target effects. Faquetti et al. [84] applied AI-driven ligand similarity analysis to uncover previously unknown interactions of 2 JAK inhibitors—baricitinib and tofacitinib—with coagulation pathways [84]. Furthermore, Datta et al. [85] used AI to analyze data from 397,064 hospitalizations in a retrospective cohort study, revealing that ondansetron, an antinausea drug, reduced hospital-acquired VTE risk by up to 11%, comparable with aspirin’s 15.5% reduction and thus supporting its potential for drug repurposing. Although not externally validated, this study combined logistic regression with input from domain experts to remove likely surrogate markers or confounding variables. Results obtained for ondansetron were consistent when repeated in a cancer subpopulation analysis and appeared to show increased ondansetron dosage was associated with higher risk reduction [85]. AI is also improving drug evaluation. Cosín-Sales et al. [86] leveraged NLP and ML to analyze real-world data from 44,292 EHRs and showed direct oral anticoagulants provided superior safety and efficacy compared to vitamin K antagonists in patients with AF, with lower incidences of thrombotic events, hospital mortality, and bleeding events. An in vitro study by Kamola et al. [87] used ML-based segmentation to classify thrombus images in flow chamber assays, improving the precision of in vitro antithrombotic drug evaluation.

## 4 Discussion

AI has the potential to enhance disease diagnostics, laboratory efficiency, risk stratification, and therapeutic decision making. In diagnostics, AI improves detection of thrombotic and bleeding events, allows for early diagnosis, and enhances access in resource-limited settings. It also offers alternatives to conventional diagnostic algorithms and assays. In laboratory medicine, AI reduces preanalytical and analytical errors and augments assay interpretation. For risk stratification, AI models can aid patient counseling, support shared decision making, and allow dynamic risk assessment. It also serves as a clinical decision-support tool, reducing clinicians’ cognitive burden. Beyond direct care, AI contributes to drug development and facilitates patient engagement.

However, despite these advances, several limitations in accuracy and clinical efficacy were noted. AI-driven software may be less sensitive in identifying certain features in imaging. For example, an AI-driven software for detecting stroke had a lower sensitivity for thrombotic occlusions despite high specificity in detecting intracerebral hemorrhage [9]. AI performance is also not universally accurate across all cohorts, with some models showing inconsistent results in bleeding risk assessment in cancer-associated thrombosis [55,56] and reduced performance in external validation cohorts [58]. These observations highlight the limitations of generalizability and underscore the need for prospective, multicenter validation.

**Figure 4:**
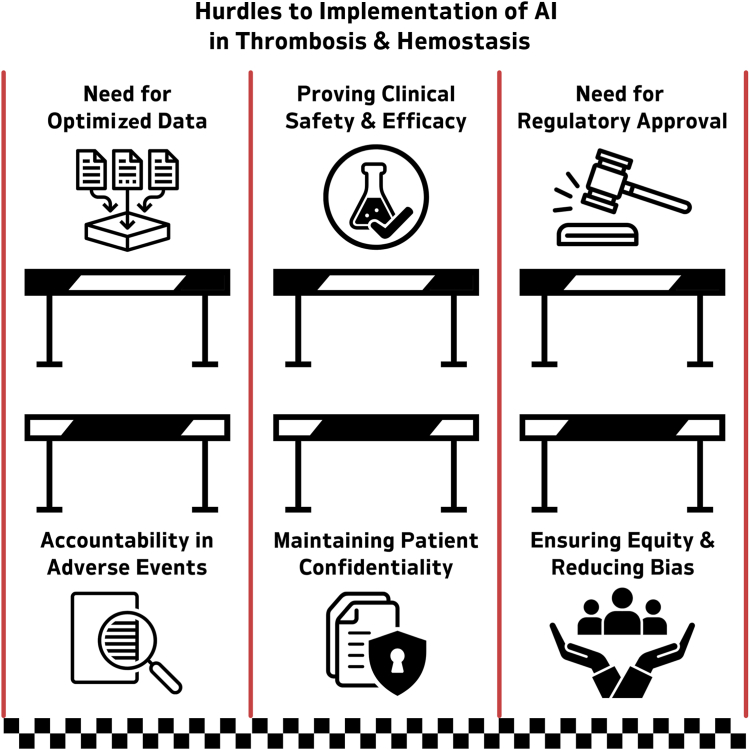
Hurdles to implementation of artificial intelligence (AI).

Beyond performance limitations, there remain significant barriers to widespread clinical implementation, including technical, regulatory, and ethical challenges (Figure 4). From a technical standpoint, the choice of AI model and the quality of training data are critical. Simpler models with fewer variables may perform comparably with complex black box models, while being more robust in the face of missing or inconsistent data [88,89]. Data handling methods such as undersampling or oversampling can introduce bias or noise, and rare events like PE may be underrepresented. Razzaq et al. [74] demonstrated that their algorithm misclassified several PE cases in women on oral contraceptives. Another example comes from an AI design challenge where participants unknowingly used warfarin dose to predict VTE—despite warfarin being initiated only after VTE diagnosis—underscoring risks of spurious correlations in unsupervised ML models [90].

From a regulatory standpoint, early steps such as the US Food and Drug Administration’s recognition of software as a Medical Device [91] are encouraging. However, overseeing continuously learning models remains a key challenge. Ensuring models are version controlled, interpretable, and auditable is essential for regulatory accountability. Ethically, ensuring privacy during AI model training is essential. Techniques such as federated learning and differential privacy allow for decentralized training without direct data sharing [92]. Another critical concern is algorithmic fairness. Without representative datasets and robust monitoring postdeployment, AI risks amplifying disparities in care [93,94]. Building clinical trust is also vital. Many AI systems, especially deep learning models, lack transparency in decision making. Research into explainable AI [95] and domain-specific interpretable models [96] is attempting to bridge this gap, but these approaches often come with trade-offs in predictive performance.

**Figure 5:**
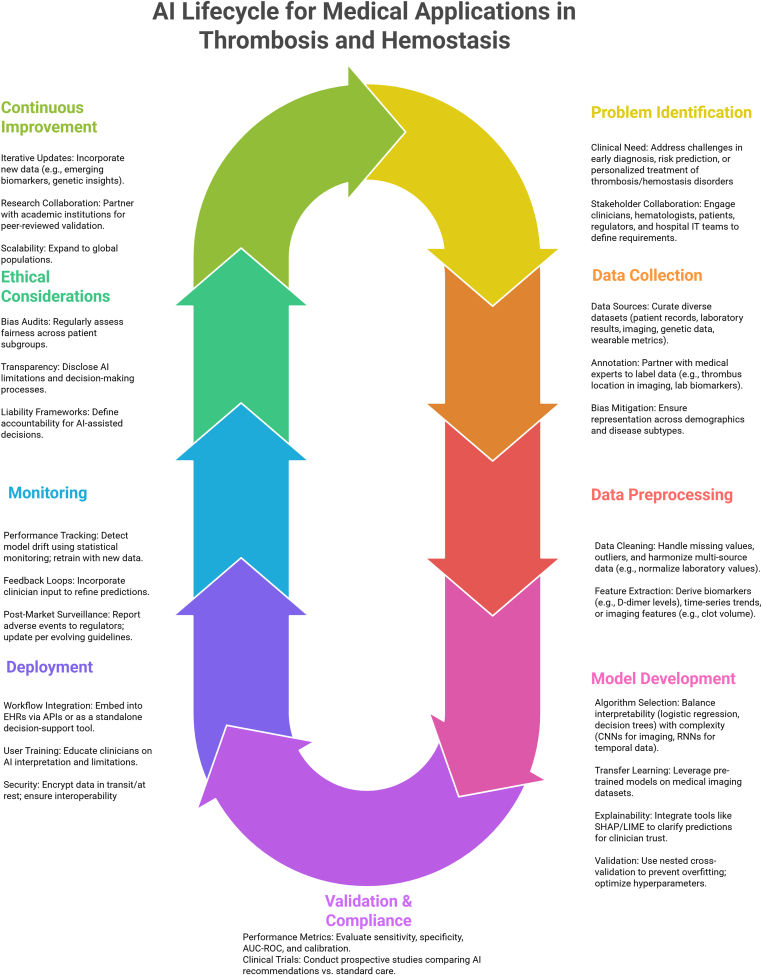
Artificial intelligence (AI) lifecycle for medical applications in thrombosis and hemostasis. AUC, area under the curve; EHR, electronic health record; IT, information technology; ROC, receiver operating characteristic.

To address such considerations, a structured AI lifecycle [97] (Figure 5) has been proposed to guide responsible deployment. This begins with clinical problem identification, followed by data collection and harmonization. Model development should include algorithm selection that balances performance with interpretability. Validation is essential to ensure clinical safety and regulatory compliance. After deployment, clinician training is necessary to support correct interpretation of outputs. Continuous monitoring is required to detect model drift, and ethical oversight should be maintained throughout. In the event of adverse outcomes, accountability frameworks and mitigation strategies must be clearly defined.

Looking ahead, the future of AI in hemostasis and thrombosis will depend on advancements in explainable AI, multimodal data integration, and real-world validation. Future research should focus on evaluating AI in large, diverse datasets, and external validation to improve model accuracy and generalisability. In addition, exploring methods such as federated learning offer potential for multi-institutional model training while preserving data privacy. Research efforts should also focus on developing interpretable, clinically relevant models that can be integrated into existing decision-making workflows. The use of AI to enhance patient engagement, education, and accessibility to health care resources is another area to be explored. Ultimately, future success will depend not just on technological innovation, but on building AI systems that are safe, equitable, and truly aligned with clinical needs.

## 5 Conclusion

Early clinical integration of AI into hemostasis and thrombosis care has clear benefits, but broader adoption requires key actions: to validate models externally in diverse, real-world settings; develop explainable AI to foster clinician trust and accountability; and strengthen ethical and regulatory frameworks to safeguard data privacy, reduce bias, and ensure equitable access. Ultimately, success will depend on balancing technological ambition with rigorous scientific scrutiny—ensuring AI not only augments human expertise but also elevates standards of care globally.
