# Supplementary material for: Artificial intelligence in clinical thrombosis and hemostasis: A review
Source: Res Pract Thromb Haemost. 2025 Jul 24;9(5):102984. doi: 10.1016/j.rpth.2025.102984 (PMC12362677; doi:10.1016/j.rpth.2025.102984)
Supplement: Supplementary Material [file mmc1.docx]

#### **Appendix**

Search Strategy………………………………………………………………………………………………………………………………Page 1

Disease Detection and Diagnosis………………………………………………………………………………………………………….Page 4

Personalized Medicine………………………………………………………………………………………………………………………Page 14

Improving Patient Education………………………………………………………………………………………………………………..Page 31

Streamlining Laboratory Testing……………………………………………………………………………………………………………Page 33

Risk Assessment and Stratification………………………………………………………………………………………………………..Page 38

Accelerating Drug Development……………………………………………………………………………………………………………Page 87

Others…………………………………………………………………………………………………………………………………………Page 98

**Search strategy**

**P:** hemostasis and thrombosis

**E:** AI-based methods or tools

**O:** diagnosis, mortality, bleeding, thrombotic complications, other complications

Initial search up until 27/8/24

Extended search until 15/2/25

**Pubmed**

| **No.** | **Query** | **Result** |
| --- | --- | --- |
| 1 | Artificial Intelligence [MeSH] OR Artificial intelligence [Title/Abstract] OR Machine learning [Title/Abstract] OR Deep learning [Title/Abstract] OR Neural network*[Title/Abstract] | 364,060 |
| 2 | embol* [Title/Abstract] OR thrombosis [Title/Abstract] OR thrombus [Title/Abstract] OR thrombi [Title/Abstract] OR thrombotic [Title/Abstract] OR thromboembol* [Title/Abstract] OR clotting [Title/Abstract] | 439,527 |
| 3 | hemostasis [Title/Abstract] OR haemostasis [Title/Abstract] OR bleed* [Title/Abstract] OR hemorrhag* [Title/Abstract] OR haemorrhag* [Title/Abstract] OR coagulopath* [Title/Abstract] OR hemophilia [Title/abstract] OR haemophilia [Title/abstract] | 594,441 |
| 4 | #1 AND #2 AND #3 | 190 |
| 5 | Extended search (Sept 2024 - 15 Feb 2025) | **31** |

**Embase**

| **No.** | **Query** | **Results** |
| --- | --- | --- |
| 1. | ‘Artificial intelligence’/exp OR ‘Machine learning’/exp OR ‘artificial intelligence’:ti,ab OR ‘machine learning’:ti,ab OR ‘deep learning’:ti,ab or ‘neural network’:ti,ab | 592,397 |
| 2. | embol*:ti,ab OR thrombosis:ti,ab OR thrombus:ti,ab OR thrombi:ti,ab OR thrombotic:ti,ab OR thromboembol*:ti,ab | 625,305 |
| 3. | hemostasis:ti,ab OR haemostasis:ti,ab OR hemostatic*:ti,ab OR haemostatic*:ti,ab OR bleed*:ti,ab or hemorrhag*:ti,ab OR haemorrhag*:ti,ab OR coagulopath*:ti,ab | 882,503 |
| 4. | #1 AND #2 AND #3 | 469 |
| 5. | Extended search (Sept 2024 - 15 Feb 2025) | **42** |

**Scopus**

| **No.** | **Query** | **Results** |
| --- | --- | --- |
| 1. | TITLE-ABS (“artificial intelligence” OR “machine learning” OR “deep learning” OR “neural networks”) | 1,593,349 |
| 2. | TITLE-ABS (embol* OR thrombosis OR thrombus OR thrombi thrombotic OR thromboembol*) | 57,652 |
| 3 | TITLE-ABS (hemostasis OR haemostasis OR hemostatic OR haemostatic OR bleed* OR hemorrhag* OR haemorrhag* OR coagulopath*) | 702,019 |
| 4. | #1 AND (#2 AND #3) | 15 |
| 5. | Extended search (Sept 2024 - 15 Feb 2025) | **5** |

| **Category** | **No of Articles** |
| --- | --- |
| Disease Detection and Diagnosis | 14 |
| Personalized Medicine | 16 |
| Improving Patient Education | 2 |
| Streamlining Laboratory Testing | 5 |
| Risk Assessment and Stratification | 38 |
| Accelerating Drug Development | 9 |
| Others | 3 |
| **Total** | **96** |

#### **Disease Detection and Diagnosis**

| **No.** | **Author** | **Title** | **Objective** | **Artificial Intelligence Model/Algorithm** | **Training set size** | **Summary** |
| --- | --- | --- | --- | --- | --- | --- |
| 1. | Barash 2024 | Artificial Intelligence for Identification of Images with Active Bleeding in Mesenteric and Celiac Arteries Angiography. | Identify active bleeding in digital subtraction angiography images for upper gastrointestinal bleeding. | Convolutional Neural Network (CNN) | 587 | This study developed and tested an AI model to detect active bleeding (extravasation) in DSA images from 142 patients with acute nonvariceal UGIB. Using the EfficientNet-B5 architecture, the model achieved 85% AUC and 77.3% accuracy in validation. While showing promise for assisting radiologists, the study acknowledges limitations including its retrospective, single-center design, exclusion of motion artifacts, and lack of external validation, highlighting the need for further research. |
| 2. | De Laat-Kremers 2024 | A thrombin-driven neural net diagnoses the antiphospholipid syndrome without the need for interruption of anticoagulation | To diagnose APS in patients who underwent vitamin K antagonist (VKA) treatment | Artificial Neural Network (ANN) | 112 | This study presents a neural network (NN) model that diagnoses antiphospholipid syndrome (APS) in patients on vitamin K antagonist (VKA) anticoagulation without requiring therapy interruption. By analyzing thrombin generation (TG) and thrombin dynamics (TD) parameters, the NN achieved 92% accuracy, 88% sensitivity, and 94% specificity. Traditional APS testing is challenging in anticoagulated patients, but this AI-driven method provides a reliable alternative. Further clinical validation is needed before widespread use. |
| 3. | Nilius 2022 | A machine-learning model for reducing misdiagnosis in heparin-induced thrombocytopenia: A prospective, multicenter, observational study. | To develop a more accurate and user-friendly diagnostic tool for heparin-induced thrombocytopenia (HIT) | Support Vector Machine (SVM)  Gradient Boosting Machine (GBM) | 1045 | The study develops TORADI-HIT, a machine-learning model to improve heparin-induced thrombocytopenia (HIT) diagnosis. Tested on 1,393 patients from 10 study centers, it achieved high accuracy (AUROC = 0.99), reducing false negatives (up to 66.7%) and false positives (up to 68.5%) compared to standard methods. The model is accessible online (<https://toradi-hit.org>) and aims to enhance diagnostic precision and patient care    Overall, the prediction model comprises six items that are easy to retrieve in clinical practice. The model outperformed the conventional 4Ts score + immunoassay approach. |
| 4. | Yoon 2018 | Machine learning-based diagnosis for disseminated intravascular coagulation (DIC): Development, external validation, and comparison to scoring systems | To optimize the use of clinical and laboratory parameters for the diagnosis of disseminated intravascular coagulation (DIC) | Artificial Neural Network (ANN) | 525 | Diagnosing DIC is difficult due to non-specific biomarkers and limitations of current scoring systems. This study developed an artificial neural network (ANN) using 32 clinical and lab parameters from 656 cases, with external validation on 217 cases. The ANN outperformed traditional DIC scoring systems (higher AUC) and revealed different parameter importance. The findings suggest ML can improve DIC diagnosis and support clinical decisions, pending further validation. |
| 5. | Mallon 2023 | Real-world evaluation of Brainomix e-Stroke software. | Interpretation of CT imaging for patients with suspected acute ischaemic stroke. | Not specified | Not specified | This study evaluated the performance of Brainomix e-Stroke, an AI-based decision support tool, in interpreting CT imaging for acute ischemic stroke in 551 patients. The tool successfully processed 97.2% of studies in an average of 4 minutes. It had high accuracy for detecting acute hemorrhage (97.8%) and large vessel occlusions (91.5%), but lower accuracy for identifying hyperdense thrombus (69.1%) and acute middle cerebral artery ischaemia (77.0%). The e-CTP's core infarct and ischemic penumbra volumes strongly correlated with those from another software.    It was able to classify the ASPECTS score with accuracy of 77% and only 3 (0.6%) patients were misclassified as ineligible for mechanical thrombectomy based on an ASPECTS of <6. However, it had a large number of false positives when identifying hyperdense vessels due to acute thrombus.  AI Identifying occlusions on CT angiogram had high specificity (93.6%) but lower sensitivity (77.6%). It also had significantly more false negatives for medium vessel occlusions, with sensitivity of 65% for large and medium vessel occlusions, hence specialist review of the CT angiogram is still needed |
| 6. | Seo 2023 | Artificial intelligence-based iliofemoral deep venous thrombosis detection using a clinical approach. | Detection of iliofemoral deep venous thrombosis (DVT) on computed tomography angiography (CTA) of the lower extremities | Convolutional Neural Network-based RetinaNet | 114 | This study used AI (specifically, CNNs) to try and improve the detection of iliofemoral deep vein thrombosis (DVT) in lower extremity computed tomography angiography (LECTA) images. It was a Retrospective analysis of 190 LECTA scans (95 with DVT, 95 without), with Preprocessing of images to enhance vessel contrast. This AI system has the potential to assist radiologists in diagnosing DVT, improving efficiency. however, it needs improvement in the classification stage to reduce false positives. |
| 7. | Kainz 2021 | Non-invasive diagnosis of deep vein thrombosis from ultrasound imaging with machine learning. | Diagnosis of DVT | CNN | 255 | The study developed an ML algorithm that can distinguish between patients with and without DVT based on ultrasound image analysis. The algorithm achieved high sensitivity (0.82-0.94) and specificity (0.70-0.82) in detecting DVT. It demonstrated a very high negative predictive value (NPV) (0.99-1.00). |
| 8. | Batra 2022 | Detection of Incidental Pulmonary Embolism on Conventional Contrast-Enhanced Chest CT: Comparison of an Artificial Intelligence Algorithm and Clinical Reports. | Detecting incidental pulmonary embolism (iPE) on conventional contrast-enhanced chest CT examinations. | Natural Language Processing (NLP) | Not specified | This study evaluated the performance of an artificial intelligence (AI) algorithm for detecting incidental pulmonary embolism (iPE) on standard contrast-enhanced chest CT scans, which are not specifically designed for PE detection. This was a retrospective study of 3003 chest CT scans from 2555 patients.    For iPE detection, AI had a high NPV of 99.8%, which was not significantly different from the NPV of 99.9% for clinical reports. Nonetheless, of the 40 iPEs present in the study sample, seven were detected only by the clinical reports, and four were detected only by AI. AI had a moderate PPV of 86.8%, which was significantly lower than the PPV of 97.3% for clinical reports. |
| 9. | Rochefort 2015 | A novel method of adverse event detection can accurately identify venous thromboembolisms (VTEs) from narrative electronic health record data. | Identify and evaluate the accuracy of statistical NLP in detecting deep vein thrombosis (DVT) and pulmonary embolism (PE) from radiology reports | SVM + NLP | Not specified | This study evaluated the accuracy of using statistical natural language processing (NLP) to identify deep vein thrombosis (DVT) and pulmonary embolism (PE) from electronic health record (EHR) radiology reports.  Retrospective analysis of 2000 randomly sampled radiology reports from patients suspected of DVT or PE.  On manual review, 324 (16.2%) reports were DVT-positive and 154 (7.7%) were PE-positive. The best DVT model achieved an average sensitivity of 0.80 (95% CI 0.76 to 0.85), specificity of 0.98 (98% CI 0.97 to 0.99), positive predictive value (PPV) of 0.89 (95% CI 0.85 to 0.93), and an area under the curve (AUC) of 0.98 (95% CI 0.97 to 0.99). The best PE model achieved sensitivity of 0.79 (95% CI 0.73 to 0.85), specificity of 0.99 (95% CI 0.98 to 0.99), PPV of 0.84 (95% CI 0.75 to 0.92), and AUC of 0.99 (95% CI 0.98 to 1.00). |
| 10. | Pham 2014 | Natural language processing of radiology reports for the detection of thromboembolic diseases and clinically relevant incidental findings. | Detect diagnoses of thromboembolic diseases (DVT and PE) and clinically relevant incidental findings in French-language angiography and venography reports. | NLP + Naïve Bayes classifier + SVM + Maximum Entropy (MaxEnt) algorithms | 100 | This study used NLP and machine learning to detect thromboembolic disease diagnosis and incidental clinically relevant findings from angiography and venography reports written in French. 573 radiology reports were used.  The best model achieved an F measure of 0.98 for pulmonary embolism identification, 1.00 for deep vein thrombosis, and 0.80 for incidental clinically relevant findings |
| 11. | Woller 2021 | Natural Language Processing Performance for the Identification of Venous Thromboembolism in an Integrated Healthcare System. | Identifying venous thromboembolism (VTE) (including DVT and PE) in imaging reports | NLP | Not specified | This study aimed to evaluate the performance of a Natural Language Processing (NLP) tool in identifying venous thromboembolism (VTE), specifically deep vein thrombosis (DVT) and pulmonary embolism (PE), within a new electronic medical record (EMR) system. Manual chart review of the 400 charts was performed.  NLP and manual review agreed on the presence of PE in 99 of 100 cases, the presence of DVT in 96 of 100 cases, the absence of PE in 99 of 100 cases and the absence of DVT in all 100 cases. When compared with manual chart review, NLP interrogation of CUS, CTPA, CT angiography of the chest, and V/Q scan yielded a sensitivity = 93.3%, specificity = 99.6%, positive predictive value = 97.1%, and negative predictive value = 99%. |
| 12. | Pederson 2021 | Deep learning detects and visualizes bleeding events in electronic health records. | Detect and visualize bleeding events in electronic health records (EHRs) | NLP + CNN + Recurrent Neural Network (RNN) | 35900 notes | This study developed a deep learning model to automatically detect and visualize bleeding events within the unstructured text of electronic health records (EHRs). Three hundred electronic health records with International Classification of Diseases, Tenth Revision diagnosis codes for bleeding or leukemia were extracted.  On a balanced test set of 1178 sentences, the best-performing deep learning model achieved a sensitivity of 0.90, specificity of 0.90, and negative predictive value of 0.90. On a test set consisting of 700 notes, of which 49 were positive for bleeding, the model achieved a note-level sensitivity of 1.00, specificity of 0.52, and negative predictive value of 1.00. |
| 13. | Chen 2023 | Application of artificial neural network in daily prediction of bleeding in ICU patients treated with anti-thrombotic therapy. | Predicting daily bleeding risk in ICU patients receiving anti-thrombotic therapy | RNN | 800 | A prediction model was built using RNNs to predict daily bleeding risk in ICU patients on anti-thrombotic drugs. The model's performance was compared to predictions made by junior and senior clinicians. 938 patients were incorporated into the study, and at the meantime, 1000 non-bleeding patients were randomly extracted.  Junior clinicians utilized an average of 77.50 min; the senior clinicians spent an average of 53 min classifying the events. All the deep learning classifiers generated prediction results within 1 min.  The accuracy of all deep learning classifiers is above 0.7, which is significantly higher than that of senior clinicians (0.6488) and junior clinicians (0.5595) |
| 14. | De Laat-Kremers 2021 | Deciphered coagulation profile to diagnose the antiphospholipid syndrome using artificial intelligence. | Diagnosis of antiphospholipid syndrome (APS) | Neural network | 97 | This study developed a neural network that accurately diagnoses APS based on thrombin generation data. The positive predictive value ranged from 62% in the hospital controls to 91% in normal controls and the negative predictive value of the neural network ranged from 86% in the thrombosis control group to 95% in the hospital controls. The sensitivity of the neural network was higher than 90% in all control groups. |
| 15. | Nagao 2024 | Artificial Intelligence-Assisted Ultrasound Imaging in Hemophilia: Research, development, and evaluation of hemarthrosis and synovitis detection. | Detect haemarthrosis and synovitis in patients with hemophilia | CNN | 3435 | This study aimed to develop and evaluate an artificial intelligence (AI) algorithm to assist in the diagnosis of hemarthrosis (joint bleeding) and synovitis (joint inflammation) in people with hemophilia using ultrasound images. 3435 ultrasound images were used for analysis  The area under the curve for hemarthrosis detection for the elbow, knee, and ankle joints was ≥0.87 and for synovitis, it was ≥0.90. The accuracy and precision for hemarthrosis detection were ≥0.74 and ≥0.67, respectively, and those for synovitis were ≥0.83 and ≥0.74, respectively. |

#### **Personalized Medicine**

| **No.** | **Author** | **Title** | **Objective** | **Artificial Intelligence Model/Algorithm** | **Training set size** | **Summary** |
| --- | --- | --- | --- | --- | --- | --- |
| 1. | Ahmad Abdel-Hafez 2022 | Predicting Therapeutic Response to unfractionated heparin therapy: Machine learning approach. | Predict the activated partial thromboplastin time (aPTT) within 12 hours after the administration of an intravenous unfractionated heparin (UFH) bolus and maintenance dose | LightGBM + XGBoost | 3019 | This study developed and validated a machine learning model to predict a patient's aPTT level after UFH administration, aiming to improve dosing accuracy. Using EHR data from 2783 hospital admission across 5 hospitals, they trained regression and multiclassification models. The ensemble models, based on LightGBM and XGBoost, showed promising performance in both cross-validation and external validation, identifying key predictive features like baseline aPTT and time since bolus administration.    A regression model was used to predict the exact aPTT value and a multiclassification model to predict the aPTT range (subtherapeutic, therapeutic, supratherapeutic).    The regression model achieved a root mean square error (RMSE) of 31.35 in cross-validation and 30.52 in external validation. The multiclassification model achieved an accuracy of 0.599 in cross-validation and 0.568 in external validation, with good performance in identifying subtherapeutic and supratherapeutic ranges, but lower accuracy for the therapeutic range. |
| 2. | Bouchnita 2022 | Combining mathematical modeling and deep learning to make rapid and explainable predictions of the patient-specific response to anticoagulant therapy under venous flow. | Predict the patient-specific response to anticoagulant regimens and assist clinical decision-making in managing coagulopathy. | ANN | 5600 | This study presents a novel AI-driven approach to predict patient-specific responses to anticoagulant therapy. By combining computational modeling and deep learning, the researchers simulate thrombin generation and thrombus formation under anticoagulation with warfarin dabigatran and rivaroxaban. A 1D model generates a dataset of thousands of virtual patients, which is then used to train an artificial neural network (ANN). The ANN achieves 96% accuracy, making it a promising tool for personalized anticoagulant dosing |
| 3. | Falconer 2021 | Systematic review of machine learning models for personalised dosing of heparin. | Systematic review. Identify and critically appraise studies that used machine learning (ML) methods for determining the optimal dosing of unfractionated heparin (UFH) | Not applicable | Not applicable | The review examines ML models for personalized heparin dosing, analyzing eight studies. While ML shows promise, models lack external validation, have inadequate reporting of study details in enabling interpretation and  reproducibility of their findings, lacked a Feasibility and impact evaluation of their use in clinical practice and are not ready for clinical use  All studies failed to adhere to one or more criteria  recommended for model development and evaluation. This includes small sample sizes, missing data and unreported features |
| 4. | Huang 2021 | Predicting range of initial Warfarin dose based on pharmacometabolomic and genetic inputs. | Predict interindividual variation in warfarin response to suggest an initial daily dose range for warfarin therapy. | Two-dimensional linear discriminant analysis-multinomial logit model | 160 | The study integrates pharmacometabolomic and pharmacogenetic data to predict individual warfarin response and suggest an initial dose range. Using data from 160 heart valve disease patients, key biomarkers—including uridine, guanosine, VKORC1 genotype, and clinical factors—were identified. A machine learning model (IniWarD) achieved 90–91% accuracy in classifying warfarin sensitivity. IniWarD outperforms conventional genotype-based dosing, potentially reducing bleeding and thrombotic risks.  IniWarD was developed to predict the probability of patients falling into different INR ranges (low, medium, high) at various warfarin doses. This allows for personalized dose recommendations.  IniWarD provided more appropriate dose ranges, especially for patients at high risk of over- or under-coagulation. |
| 5. | Ma 2018 | Ensemble of machine learning algorithms using the stacked generalization approach to estimate the warfarin dose. | Predicting the stable therapeutic dose of warfarin. | Support vector regression + ridge regression + neural networks + gradient boosting trees | 4594 | The paper presents a stacked generalization machine learning approach to improve warfarin dose prediction, addressing the challenges of high interpatient variability. By combining multiple ML models, including support vector regression, ridge regression, neural networks, and gradient boosting trees, the study outperformed traditional multivariate linear regression (MLR) models. The proposed ensemble model showed higher accuracy, especially for Asian patients and those requiring low doses, reducing the risk of thrombosis or bleeding.  For Asians and low-dose patients, the new algorithms improved the accuracy of predicting the warfarin dose within 20% of the actual therapeutic dose by 12.7% and 13.5%, respectively |
| 6. | Petch 2024 | Optimizing warfarin dosing for patients with atrial fibrillation using machine learning | Optimize warfarin dosing and improve time in therapeutic INR range for patients with atrial fibrillation | Reinforcement learning model | 28,232 | The study develops a reinforcement learning (RL) model for personalized warfarin dosing in atrial fibrillation patients, using data from 28,232 patients. The model improves time in therapeutic INR range (TTR) and reduces stroke risk, performing on par with clinical algorithms. A 10% increase in algorithm-consistent dosing improved TTR by 6.78% and reduced adverse events by 11%. The model shows promise for clinical use, but prospective trials are needed for validation.    These results were comparable to those of a rules-based clinical algorithm used for benchmarking, for which each 10% increase in algorithm-consistent dosing independently predicted a 6.10% increase in TTR (95% CI 5.67, 6.54, *p* < 0.001) and a 10% decrease in the composite outcome (HR 0.90; 95% CI 0.83, 0.98, *p* = 0.018). |
| 7. | Gan 2025 | AI‐assisted warfarin dose optimisation with CURATE.AI for clinical impact: Retrospective data analysis. | Optimize and guide warfarin dosing | Not specified | 127 | This study evaluated CURATE.AI, an AI platform, for optimizing warfarin dosing in 127 patients. The platform used warfarin dose and INR changes to predict optimal doses and minimize adverse events like thromboembolism and bleeding. CURATE.AI outperformed other models in prediction accuracy, with negligible underprediction bias. Its predicted time in the therapeutic range was comparable to physician-guided dosing, offering a systematic approach that reduces physician guesswork. |
| 8. | Razzaq 2021 | An artificial neural network approach integrating plasma proteomics and genetic data identifies PLXNA4 as a new susceptibility locus for pulmonary embolism | Identify biomarkers associated with the risk of pulmonary embolism (PE) in patients with deep vein thrombosis (DVT) | ANN | 1,388 | This study developed an artificial neural network (ANN) model to identify biomarkers for PE risk in DVT patients using proteomic and genetic data from the MARTHA study (1,388 patients). GWAS identified PLXNA4 (rs1424597 SNP) as a PE risk gene.  In a sample of 112 COVID-19 patients known to have endotheliopathy leading to acute lung injury and an increased risk of PE, decreased PLXNA4 levels were associated (p = 0.025) with worsened respiratory function.    3 female PE patients wrongly predicted to be DVT (individuals 11, 12 and 15) were all under oral contraceptives (OC) at the time of the PE event (age 45, 35 and 53, respectively), but not individual 10 incorrectly predicted to be PE. |
| 9. | Penco 2005 | Assessment of the role of genetic polymorphism in venous thrombosis through artificial neural networks. | Assess the role of genetic polymorphisms in venous thrombosis events (VTE) | ANN | 449 | This study used artificial neural networks (ANNs) to analyze the role of genetic polymorphisms in venous thromboembolism (VTE) using data from 238 patients and 211 controls. ANNs outperformed logistic regression in predicting VTE cases vs. controls. Through ANNs models, the 62 variables related to genetic variants were first reduced to a set of 9, and then of 3 (MTHFR 677 C/T, FV arg506gln, ICAM1 gly214arg). |
| 10. | Liu 2014 | Improvement of adequate use of warfarin for the elderly using decision tree-based approaches. | Predict drug-to-drug interactions (DDIs) and improve effectiveness for warfarin | C4.5 + k-nearest neighbors (kNN) + classification and regression tree (CART) + random forest (RF) + multi-layer perceptron neural network with back-propagation (MLP) + Support vector machine (SVM) | 377 | This study investigates the use of machine learning (ML) techniques to improve warfarin dosing for elderly inpatients, addressing challenges posed by its narrow therapeutic range and high drug-to-drug interactions (DDIs). Using decision tree-based classifiers, the study evaluates their effectiveness in predicting adequate warfarin use, particularly when incorporating laboratory data and patient history. The study analyzed 288 cases with DDIs and 89 cases without DDIs to assess ML model performance.  For non-DDI patients, key predictive factors included Weight, congestive heart failure (CHF), and gender. For DDI patients they were Age, alanine aminotransferase (ALT), and warfarin dose. |
| 11. | Sharabiani 2015 | Revisiting Warfarin Dosing Using Machine Learning Techniques. | Develop a new methodology for warfarin dosing by classifying patients into two categories based on their required weekly doses (>30 mg/wk and ≤30 mg/wk) and predicting the optimal dose for each class using customized clinical regression models. | Relevance vector machines (RVM) + Clinical regression models | 2,118 | This study proposed a new methodology for warfarin dosing using a two-phase machine learning approach to improve dose prediction accuracy. The patients are initially classified into two classes. The first class contains patients who require doses of >30 mg/wk and the second class contains patients who require doses of ≤30 mg/wk. This phase is performed using relevance vector machines. In the second phase, the optimal dose for each patient is predicted by two clinical regression models that are customized for each class of patients. The prediction accuracy of the model was 11.6 in terms of root mean squared error (RMSE) and 8.4 in terms of mean absolute error (MAE). This was 15% and 5% lower than IWPC and Gage models (which are the most widely used models in practice), respectively, in terms of RMSE |
| 12. | Gordon 2021 | Using machine learning to predict anticoagulation control in atrial fibrillation: A UK clinical practice research datalink study. | Investigate the predictive performance of machine learning (ML) algorithms for estimating anticoagulation control in patients with atrial fibrillation (AF) treated with warfarin | SVM + Random forests + XGBoost + NN + LSTM | 35,479 | This study investigated the use of machine learning (ML) algorithms to predict suboptimal anticoagulation control in patients with atrial fibrillation (AF) treated with warfarin. 35,479 patients were included. Addition of time-varying data (especially prior INR measurements) to the LSTM NN improved predictive performance, plateauing at AUC of 0.830 at 30 weeks. |
| 13. | Kim 2021 | Gene Polymorphisms of the Renin-Angiotensin System and Bleeding Complications of Warfarin: Genetic-Based Machine Learning Models. | Investigate the effects of genetic variants and haplotypes in the renin-angiotensin system (RAS) on the risk of warfarin-induced bleeding complications at therapeutic international normalized ratios (INRs) | Multivariate logistic regression + Elastic net + Random forest + SVM with linear kernel + SVM with radial kernel | 142 | This study examined the impact of RAS genetic variants on warfarin-induced bleeding risk in 142 patients, identifying key genetic factors influencing complications. Logistic regression and several machine learning methods were used for bleeding prediction. The highest quartile group (≥75th percentile) of weighted risk score had approximately 12.0 times (95% CI 3.1-46.7) increased risk of bleeding, compared to the 25-75th percentile group, respectively. This study demonstrated that RAS-related polymorphisms, including the H2 haplotype of the ACE gene, could affect bleeding complications during warfarin treatment for patients with mechanical heart valves |
| 14. | Rawal 2024 | Application of machine learning approaches for predicting hemophilia a severity. | Predict the severity of Hemophilia A (HA) in female patients | LR + RF + GB + LGBM + XGB + CB | 2688 | This study used machine learning (ML) to predict the severity of Hemophilia A (HA) in female patients based on F8 gene variants. By analyzing multiple datasets and deriving the FVIII protein sequence, the ML models achieved high predictive accuracy (F1 scores between 0.88 and 0.99). The results confirm that ML can effectively predict HA severity in females, offering potential for better treatment and clinical outcomes in this often underdiagnosed group.  When we trained the model using data from male patients, the HA severity in females was predicted with high accuracy. The ROC curves (Figure 2) show that the best-performing model can distinguish between mild, moderate, and severe HA with high sensitivity (AUC = 1) and precision (AUC 0.85 to 1).  The most critical limitation of this study was that data from only 273 genotyped females with corresponding FVIII activity measurements was available to test the models’ predictive performance in females. |
| 15. | Ferreira 2023 | A graph-based machine learning framework identifies critical properties of FVIII that lead to hemophilia A. | Explore the role of each residue of the coagulation factor VIII (FVIII) protein in the pathogenesis of Hemophilia A (HA) | Graph-based Neural network | 626 | This study developed a graph-based machine learning framework to analyze the coagulation factor VIII (FVIII) protein and its role in hemophilia A (HA).The graph-based model mapped FVIII’s 3D structure, identifying residues linked to severe and mild HA cases.  The model successfully predicted activity and expression for 300+ alanine mutations, closely matching in vitro results. |
| 16. | Lopes 2021 | Prediction of hemophilia A severity using a small-input machine-learning framework. | Predict and understand how mutations in the FVIII protein affect the severity of Hemophilia A | Decision tree + random forest + SVM + Naïve Bayes + XGBoost | 443 | This study used machine learning (ML) and protein structure analysis to investigate how Factor VIII (FVIII) mutations affect Hemophilia A severity. This study designed a machine-learning framework to improve the understanding of the relationship between the protein structure and the disease severity. We verified a close agreement between in silico, in vitro and clinical data. Finally, we predicted the severity of all possible mutations in the FVIII structure - including those not yet reported in the medical literature. |

#### **Improving Patient Education**

| No. | Author | Title | Objective | Artificial Intelligence Model/Algorithm | Training set size | Summary |
| --- | --- | --- | --- | --- | --- | --- |
| 1. | Labovitz 2017 | Using artificial intelligence to reduce the risk of nonadherence in patients on anticoagulation therapy. | Evaluate the use of an artificial intelligence platform on mobile devices for measuring and improving medication adherence in stroke patients on anticoagulation therapy | NA | NA | The study evaluates an AI-based mobile platform to improve medication adherence in stroke patients on anticoagulation therapy. The AI application visually identified the patient, the medication and confirmed ingestion. A 12-week randomized trial (n = 28) compared AI-monitored patients to a control group. AI monitoring improved adherence, with 100% adherence in the AI group vs. 50% in controls (based on plasma drug levels). DOAC patients saw a 67% improvement. |
| 2. | Lewandowska 2022 | Therapeutic and technological advancements in haemophilia care: Quantum leaps forward. | Review new and emerging therapies in haemophilia, including replacement and bypassing products, digital applications, the use of big data, and personalized medicine | NA | NA | A chatbot was designed to address identified disease knowledge gaps among PwH in Senegal. This unique chatbot is designed to recognize voices speaking in French and Wolof (a common language in Senegal), providing text answers in both languages through a user-friendly interface integrated into both Android and Apple iOS devices. |

#### **Streamlining Laboratory Testing**

| No. | Author | Title | Objective | Artificial Intelligence Model/Algorithm | Training set size | Summary |
| --- | --- | --- | --- | --- | --- | --- |
| 1 | Lippi 2024 | Artificial intelligence in the pre-analytical phase: State-of-the art and future perspectives | Review paper of AI in the pre-analytical phase of laboratory medicine | NA | NA | The paper explores the role of artificial intelligence (AI) in the pre-analytical phase of laboratory medicine, an area often prone to errors.  AI aids in patient identification using biometric recognition, automated sample labeling, vein detection, and robotic blood drawing, which improves efficiency and reduces human error​. AI optimizes sample transport using pneumatic transport systems, drones, and smart blood tubes, ensuring timely and safe delivery of specimens​.  Automated systems evaluate sample quality by detecting hemolysis, clot formation, and insufficient filling, reducing diagnostic errors​. |
| 2. | Qian 2024 | Coagulo-Net: Enhancing the mathematical modeling of blood coagulation using physics-informed neural networks. | Enhance blood coagulation models using Coagulo-Net, a deep learning framework that combines deep neural networks with the mechanistic understanding of blood coagulation processes. | Physics-informed Neural network | 500,000 epochs | This research introduces Coagulo-Net, a deep learning model designed to improve mathematical models of blood coagulation. Existing coagulation models have challenges in estimating parameters due to unmeasurable variables. Coagulo-Net integrates deep neural networks with the understanding of coagulation processes to enhance model accuracy. It can infer unknown parameters and species dynamics from limited or noisy data and refine predictions by processing both synthetic and experimental data. The study demonstrates Coagulo-Net's potential to improve coagulation models, aiding in better research methodologies and the development of treatments for coagulation disorders. |
| 3. | Fang 2021 | Using machine learning to identify clotted specimens in coagulation testing. | Demonstrate the feasibility of using machine learning (ML) to automatically detect blood clots in specimens during coagulation testing. | Backpropagation neural network (BPNN) | 428 | This study explored the feasibility of using machine learning (ML) to automatically detect blood clots in coagulation test samples. 192 clotted samples and 2,889 no-clot-detected (NCD) samples were retrospectively retrieved. neural networks could identify the sample status (clotted and NCD) with areas under the ROC curves of 0.966 (95% CI, 0.958-0.974) and 0.971 (95% CI, 0.9641-0.9784), respectively. |
| 4. | Arumugam 2016 | Random Forests Are Able to Identify Differences in Clotting Dynamics from Kinetic Models of Thrombin Generation. | Improve the classification of acute coronary syndromes (ACS) from stable coronary artery disease by leveraging a more complex model of the clotting cascade | Random forest | Not specified | Differences associated with acute coronary syndromes emerge in combinations of a handful of features. For instance, concentrations of 3 chemical species, namely, active alpha-thrombin, tissue factor-factor VIIa-factor Xa ternary complex, and intrinsic tenase complex with factor X, at specific time windows, could be used to classify acute coronary syndromes to an accuracy of about 87.2%. Such a combination could be used to efficiently assay the coagulation system. |
| 5. | Zhou 2022 | A highly accurate delta check method using deep learning for detection of sample mix-up in the clinical laboratory. | Detect sample mix-ups in hematology tests | SVM + KNN + RF + LR + Native Bayesian Classifier (NBC) + Deep belief network (DBN) | Not specified | This study developed a deep learning-based delta check (DC) method to improve sample mix-up detection in hematology tests. Using a Deep Belief Network (DBN) trained on data from two hospitals, the model outperformed three comparator statistical methods, achieving 93.1% accuracy and an ROC-AUC of 0.977. |

####

#### **Risk Assessment & Stratification**

| No. | Author | Title | Objective | Artificial Intelligence Model/Algorithm | Training set size | Summary |
| --- | --- | --- | --- | --- | --- | --- |
| 1. | Abbasi 2020 | Machine learning to predict hemorrhage and thrombosis during extracorporeal membrane oxygenation. | Predicting hemorrhage and thrombosis during extracorporeal membrane oxygenation | Random forest + recursive feature elimination + decision trees +  k-nearest neighbors + logistic regression | 44 | The study uses machine learning to predict hemorrhage and thrombosis in ECMO patients Using data from 44 patients. Hemorrhage models (58–80% accuracy) outperformed thrombosis models(40–64%). ML identified novel risk factors beyond traditional methods (including ECMO configuration, cannulation strategy, and patient demographics), highlighting its potential for better ECMO risk assessment with larger datasets. |
| 2. | Abbasi 2023 | Interpretable Machine Learning-Based predictive modeling of patient outcomes following cardiac surgery. | Predicting high-risk complications—postoperative hemorrhage requiring reoperation, venous thromboembolism (VTE), and stroke—after cardiac surgery | Feedforward neural network (FNN) | 530,218 | The study applies machine learning to predict postoperative hemorrhage, VTE, and stroke in 662,772 cardiac surgery patients. The model achieved high accuracy (AUROC 0.92–0.97), with postoperative variables (e.g., mechanical ventilation, arrhythmias) being the most influential predictors. Findings suggest targeting postoperative care could reduce complication risks. |
| 3. | An 2023 | A life-threatening bleeding prediction model for immune thrombocytopenia based on personalized machine learning: a nationwide prospective cohort study | Predict critical bleeding events in patients with immune thrombocytopenia (ITP) | Logistic regression + LightGBM + adaptive boosting + multilayer perceptron + support vector machine + RF + XGBoost | 1494 | The study develops a machine-learning model to predict life-threatening bleeding in ITP using nationwide data. The random forest model achieved high accuracy (AUC = 0.89 retrospective, 0.82 prospective inpatient, 0.74 outpatient),A web-based tool was created for real-time clinical use.    Age, PLT count, infection, uncontrolled diabetes mellitus, and ITP type were identified as predictors through the models. The largest contributor to the model output was the initial diagnosis of PLT count, which reflected the severity of ITP |
| 4. | Bouchnita 2023 | Combining Computational Modelling and Machine Learning to Identify COVID-19 Patients with a High Thromboembolism Risk | Predict thromboembolism risk in COVID-19 patients | Neural network + SVM | 2400 | The study combines computational modeling and machine learning to predict thromboembolism risk in COVID-19 patients. A mathematical model simulates thrombus formation and thrombin generation, showing that COVID-19 autoantibodies increase clotting risk. A machine learning algorithm, trained on simulated patient data, achieved 95% accuracy in identifying high-risk patients. The findings suggest this approach could support early thrombosis detection and intervention in COVID-19 cases. |
| 5. | De Laat-Kremers 2022 | Coagulation parameters predict COVID-19-related thrombosis in a neural network with a positive predictive value of 98% | Predicting thrombosis in COVID-19 patients | Neural network | 133 | The study develops a neural network model to predict COVID-19-related thrombosis using coagulation parameters. Based on data from 149 hospitalized COVID-19 patients, the model achieved 98–100% positive predictive value, identifying key risk factors like C-reactive protein, thrombin generation parameters, sex. |
| 6. | Fresard 2020 | Multi-Objective optimization for personalized prediction of venous thromboembolism in ovarian cancer patients | Predicting thrombotic events in ovarian cancer patients | KNN | Not specified | This study develops machine learning models to predict thrombotic risk in ovarian cancer patients, addressing the challenge of imbalanced datasets. Using data from 121 high-grade serous ovarian carcinoma patients, the approach employs cost-sensitive classification and multi-objective optimization to create personalized risk models.  Traditional risk models like the Khorana score have high false positive rates. This study used machine learning, specifically cost-sensitive K-Nearest Neighbors and multi-objective optimization, to improve prediction accuracy for VTE/DVT in an imbalanced dataset. New models showed improved performance over traditional models, balancing sensitivity and specificity better. |
| 7. | Goto 2020 | New artificial intelligence prediction model using serial prothrombin time international normalized ratio measurements in atrial fibrillation patients on vitamin K antagonists: GARFIELD-AF. | Predict clinical outcomes (major bleeding, stroke/systemic embolism, and death) up to 1 year in atrial fibrillation (AF) patients treated with vitamin K antagonists (VKAs). | 1D CNN + LSTM | 2,230 | This study developed and validated an AI model to predict major bleeding, stroke/systemic embolism (SE), and all-cause death in atrial fibrillation (AF) patients treated with vitamin K antagonists (VKAs) between months 2 and 12. The model used only serial prothrombin time international normalized ratio (PT-INR) measurements taken within the first 30 days of treatment, a novel approach compared to traditional models that use single time-point data. Using data from the GARFIELD-AF registry, the AI model, based on recurrent neural networks, showed better predictive accuracy than time in therapeutic range (TTR), particularly for major bleeding and especially in the early phase of VKA initiation. While the model has limitations, it suggests that AI can extract valuable predictive information from early PT-INR patterns  They may discriminate patients who are unsuitable for VKA therapy and suggest switching them to NOACs, which are associated with lower bleeding risk compared with VKA. |
| 8. | Grdinic 2024 | Developing a machine learning model for bleeding prediction in patients with cancer-associated thrombosis receiving anticoagulation therapy. | Predicting bleeding in cancer-associated thrombosis (CAT) patients and compare its performance to the existing CAT-BLEED score. | Logistic regression + random forest + XGBoost | 1080 | This study developed a machine learning model to predict bleeding in cancer-associated thrombosis (CAT) patients. Using data from 1080 patients, they compared the predictive performance of the CAT-BLEED score with several machine learning algorithms. Lasso logistic regression and XGBoost outperformed the CAT-BLEED score in predicting bleeding at both 90-day and 365-day intervals.  ML models have better performance than that of the CAT-BLEED score (AUROC, 0.65 ± 0.06 vs 0.48 ± 0.13).  However, while the AUROC was improved, the precision, especially when targeting high recall (90%), remained a challenge, indicating the models still struggled to accurately identify a sufficient number of patients at risk for bleeding.    With a recall of 0.9, the precision of XGBoost was 0.11 in detecting major bleeding or CRNMB 1 to 90 days after VTE. This is only slightly better than chance, as the incidence of bleeding in this experiment was 8%. |
| 9. | Herrin 2021 | Comparative effectiveness of machine learning approaches for predicting gastrointestinal bleeds in patients receiving antithrombotic treatment | Compare the predictive performance of machine learning models with the HAS-BLED risk score in predicting gastrointestinal bleeding (GIB) in patients prescribed antithrombotic treatment | Regularized Cox proportional hazards regression (RegCox) + random survival forests (RSF) + XGBoost | 105 837 | This study compared machine learning models with the HAS-BLED score for predicting gastrointestinal bleeding (GIB) in patients starting antithrombotic treatment. Using a large claims database (OptumLabs), they analyzed over 300,000 patients, finding that regularized Cox regression (RegCox) and XGBoost outperformed HAS-BLED in predicting GIB at 6 and 12 months. All models had limitations in positive predictive value.    In the validation data set, the HAS-BLED model had an AUC of 0.60 for predicting GIB at 6 months and 0.59 at 12 months. The RegCox model performed the best in the validation set, with an AUC of 0.67 at 6 months and 0.66 at 12 months. |
| 10. | Hsu 2023 | Application of machine learning to predict postoperative gastrointestinal bleed in bariatric surgery. | Predicting postoperative gastrointestinal bleed in bariatric surgery | Random forest + gradient boosting + neural network + logistic regression | 127,967 | This study develops machine learning (ML) models to predict postoperative GIB after bariatric surgery, using data from 159,959 patients. ML models (random forest, gradient boosting, neural networks) outperformed logistic regression (AUROC 0.764 for RF vs. 0.709 for LR), with random forest performing best. Key predictors included surgery type, pre-op hematocrit, age, procedure duration, and creatinine levels. ML-based risk prediction can aid surgeons in decision-making, though more interpretable models are needed. |
| 11. | Huang 2023 | Effectiveness of an artificial intelligence clinical assistant decision support system to improve the incidence of hospital-associated venous thromboembolism: a prospective, randomised controlled study | Improving thromboprophylaxis practices and reducing hospital-associated venous thromboembolism (HA-VTE) in hospitalized patients. | NLP | Not specified | The study evaluates an AI-driven Clinical Decision Support System (AI-CDSS) for preventing hospital-associated venous thromboembolism (HA-VTE) in a randomized controlled trial of 19,785 hospitalized patients. AI-CDSS reduced HA-VTE incidence by 46%, increased mechanical prophylaxis by 24%, and improved drug use intensity by 9.72%. However, it did not significantly impact overall prophylaxis rates. The findings suggest AI-CDSS can enhance VTE prevention, but further research is needed to optimize implementation. |
| 12. | Liu 2023 | Ten-Year multicenter retrospective study utilizing machine learning algorithms to identify patients at high risk of venous thromboembolism after radical gastrectomy. | Predict the onset of venous thromboembolism (VTE) in patients undergoing surgery for gastric cancer | XGBoost + random forest + SVM + KNN | 611 | The study develops a machine learning model to predict venous thromboembolism (VTE) after radical gastrectomy using data from 1,239 gastric cancer patients. Among four models tested (XGBoost, random forest, SVM, KNN), XGBoost performed best (AUC = 0.989 training, 0.912 validation, 0.85 external validation). Key risk factors included BMI, adjuvant radiotherapy/chemotherapy, surgical duration, intraoperative bleeding, and central venous catheter use. |
| 13. | Mittman 2024 | Development and internal validation of the Cleveland Clinic Bleeding Model to predict major bleeding risk at admission in medical inpatients. | Predicting major in-hospital bleeding in acutely ill medical patients | LASSO logistic regression | 32, 420 | This study developed a novel risk assessment model (CCBM) for predicting major bleeding in hospitalized patients, comparing it with the existing IMPROVE model. The cohort consisted of over 46,314 patients from 10 Cleveland Clinic hospitals. The model showed good calibration and discrimination, outperforming IMPROVE, with a significantly higher area under the curve (AUC) of 0.86 compared to IMPROVE’s 0.72.  CCBM had similar sensitivity but categorized fewer patients as high-risk than IMPROVE, with those categorised as high risk having higher bleeding risk.  The findings suggest CCBM may improve clinician decision-making for VTE prophylaxis, as it allows for more patients to receive prophylaxis without increasing their bleeding risk. However, the model still requires external validation    Limitations - a small number of outcomes to develop and test our model. Our cohort’s major bleeding rate was 0.58%, less than the total bleeding rate in the IMPROVE cohort and external validation studies, which included nonmajor bleeding. |
| 14. | Mora 2023 | Machine learning to predict major bleeding during anticoagulation for venous thromboembolism: possibilities and limitations. | Predicting major bleeding during anticoagulation for venous thromboembolism | SVM + KNN + NN + Decision tree + XGBoost | 34,710 | This study evaluates the use of machine learning (ML) to predict major bleeding in patients with venous thromboembolism (VTE) during the first 3 months of anticoagulation therapy. Traditional risk scores (RIETE and VTE-BLEED) have low predictive value, especially for high-risk patients. The study found that the XGBoost ML algorithm performed better than traditional scores in identifying patients at high risk for MB, with high sensitivity (91.2%), specificity (91%), PPV (90.4%), and NPV (90.3%).  However, its performance dropped in an external validation cohort (COMMAND-VTE) and was worse than that of the RIETE score and VTE BLEED score. In that cohort, the F1 value was 5.2% for the ML, for the RIETE score was 17.3% and for the VTE-BLEED score 9.75%. This may be due to missing data (it lacked 14 variables that were part of the XGBoost model) and differences in patient characteristics. |
| 15. | Nopp 2022 | Bleeding risk Assessment in End-Stage Kidney Disease: Validation of existing risk scores and evaluation of a Machine Learning-Based approach | Predicting major bleeding risk in patients with end-stage kidney disease (ESKD) on hemodialysis (HD). | KNN + decision tree + random forest + neural network | Not specified | This study explored bleeding risk prediction in patients with ESKD on HD. Despite using both traditional bleeding risk scores (like HAS-BLED) and machine learning models, neither was effective in predicting major bleeding events.  None of the seven bleeding risk assessment tools showed a C-statistic above 0.60, indicating poor performance to identify patients at low or high risk for bleeding.  The four commonly used machine learning models, which were based on 25 features comprising patient demographics, clinical characteristics, and laboratory measurements (did not predict major bleeding within 24 months better than chance. |
| 16. | Nazarian 2024 | Development and validation of machine learning models to predict the need for haemostatic therapy in acute upper gastrointestinal bleeding. | Predict the need for haemostatic therapy (via endoscopy, radiology, or surgery) in patients with acute upper gastrointestinal bleeding (AUGIB) | Random forest + extra tree + gradient boost + KNN + decision tree + ensemble learning | 776 | The study aimed to develop and validate machine learning models to predict the need for haemostatic therapy (endoscopy, radiology, or surgery) in acute upper gastrointestinal bleeding (AUGIB). Retrospective cohort study analyzing 970 patients admitted with AUGIB from 2015 to 2020. Random Forest Model had higher AUROC than traditional Glasgow-Blatchford Score (GBS). For ML model - AUROC = 0.84 (0.80–0.87) vs for GBS: 0.75 (0.72–0.78), p < 0.001). |
| 17. | Fard 2024 | A deep learning approach to predict bleeding risk over time in patients on extended anticoagulation therapy. | Prediction of major bleeding in patients on extended anticoagulation therapy | ANN + RNN | 1,779 | This study developed a deep learning model to predict major bleeding in patients on extended anticoagulation therapy by incorporating time-series follow-up data collected every 6 months. Using data from 2,542 patients over eight years, the best-performing model—an ensemble of feedforward and recurrent neural networks—achieved 61% sensitivity and 82% specificity, outperforming the HAS BLED score.  The Precision of this model was 13%    At their standard threshold, the HAS-BLED achieved a precision of 19%. However, the HAS-BLED’s high precision had come at the cost of lower sensitivity of 33%. |
| 18. | Shohat 2023 | Using machine learning to predict venous thromboembolism and major bleeding events following total joint arthroplasty | Predict venous thromboembolism and major bleeding events in patients who have undergone total joint arthroplasty | Random forest + LASSO + Gradient Boosting Trees + SVM | 25,174 | This study developed and validated a machine learning model to predict venous thromboembolism (VTE) and major bleeding events (MBE) in total joint arthroplasty (TJA) patients, analyzing 35,963 cases from 2009–2020.    The Gradient Boosting Trees model best predicted VTE (AUC-ROC: 0.77), while LASSO regression was optimal for MBE (AUC-ROC: 0.80). The model provides a personalized risk assessment tool, aiding in balanced prophylaxis decisions for TJA patients.    However, “Black box” analyses as the ones used in this study are not easily interpretable and it remains unclear how the model predicts outcome. While relative importance and cross validation shed some light on what the model relies on more heavily, we are still left with some uncertainty. |
| 19. | Truong 2024 | Development and Validation of Machine Learning Algorithms to Predict 1-Year Ischemic Stroke and Bleeding Events in Patients with Atrial Fibrillation and Cancer. | Predicting ischemic stroke and major bleeding in patients with atrial fibrillation and cancer | Elastic net logistic regression + random forest + SVM + XGBoost + Neural network | Not specified  (Total cohort 18,388 patients) | This study used machine learning to predict stroke and bleeding risks in atrial fibrillation patients with cancer. Using SEER-Medicare data, five ML models were tested.  The final cohort consisted of 18,388 patients, of whom 523 (2.84%) had ischemic stroke and 221 (1.20%) had major bleeding within one year after AFib diagnosis  Stroke Prediction: Random Forest (RF) performed best (AUC 0.916, sensitivity (0.868), specificity (0.801), and F2 score (0.375).) of all ML models and also outperformed CHA₂DS₂-VASc. Although CHA_2_DS_2_-VASc score showed a higher sensitivity (0.829) compared to other ML models (except for RF), its specificity was low (0.268).  Key factors included socioeconomic status, time from cancer diagnosis to AFib onset, history of stroke, and concomitant use of ACE inhibitors or ARBs.  Bleeding Prediction: All models, including HAS-BLED, performed poorly (all AUC < 0.7 for ML models, for HAS -BLED sensitivity = 0.052).), highlighting the complexity of bleeding risks.  Conclusion: ML, especially RF, improves stroke risk prediction in AFib cancer patients but needs refinement for bleeding risk. |
| 20. | Umemura 2024 | A machine learning model for early and accurate prediction of overt disseminated intravascular coagulation before its progression to an overt stage. | Prediction of overt disseminated intravascular coagulation (DIC) in septic patients | LightGBM | 100 | This single-center, retrospective study developed a machine learning model to predict the onset of disseminated intravascular coagulation (DIC) in septic patients *before* it reaches the overt stage. Using data from 912 septic patients (139 of whom developed overt DIC), the model, based on Light Gradient Boosted Machine (LightGBM), achieved higher accuracy (84.4% sensitivity, 87.5% specificity, 0.867 AUROC in the test cohort) than traditional scoring systems (JAAM DIC and ISTH SIC criteria) for predicting the onset of overt DIC within 7 days after the sepsis diagnosis. The model identified circulatory parameters like urine output and lactate levels and the cardiovascular component of the SOFA score as key predictors. While acknowledging limitations like its single-center and retrospective nature, the study suggests machine learning can improve early DIC prediction |
| 21. | Van De Sande 2020 | Predicting thromboembolic complications in COVID-19 ICU patients using machine learning. | Early identification of COVID-19 patients at risk of thromboembolic complications (TCs) during ICU admission | Decision tree | 82 | This single-center retrospective study developed a decision tree model to predict thromboembolic complications (TCs) in critically ill COVID-19 ICU patients. Analyzing data from 108 patients (43 with TCs), the model, based on LDH, leukocytes, bicarbonate, and albumin levels, achieved an AUROC of 0.76, 73% sensitivity, and 73% specificity in the test dataset. The model showed potential for identifying high-risk patients, with a sensitivity ≥74% already starting on day 4 before a diagnosis of a TC. While acknowledging limitations like the small sample size and retrospective design, the authors suggest this easily implementable model, using routine lab values, could improve risk stratification and enable earlier intervention. |
| 22. | Zanaty 2020 | Predicting chronic subdural hematoma recurrence and stroke outcomes while withholding antiplatelet and anticoagulant agents. | Identify predictors for the recurrence of chronic subdural hematoma (cSDH), thromboembolism (TEE), hospital stay, and mortality in patients, and to determine the optimal window for resuming oral anticoagulation (OAC) or antiplatelet drugs (APD) after cSDH. | Reinforcement fine tuning (RFT) + Multiple linear regression (MLR) | 477 | This study of 596 cSDH patients aimed to predict key outcomes and optimize the timing of resuming blood thinners after surgery. They found that chronic kidney disease and platelet dysfunction, smoking OAC use increased the risk of cSDH recurrence.  The lowest risk of recurrence was the resumption of OAC between 2 and 20 days, with a slight increase after 20 days. The chance of developing TEEs is small and exhibits a small increase with longer time off APD or OAC. We find the optimal time of resuming OAC to be after 2 days but before 21 days as these patients had the lowest recurrence of bleeding associated with a low risk of stroke.  Machine learning successfully predicted recurrence (an accuracy of 93%, precision of 0.84 (specificity = 84%) and recall of 0.80 (sensitivity = 80%))  Unable to use ML model on TEEs (due to low event numbers) and poor accuracy in predicting hospital stay (due to many influencing factors).    Recurrence rate: 22.17%, TEE occured in 0.9%, mortality: 14.78%. |
| 23. | Zhao 2024 | Harnessing risk assessment for thrombosis and bleeding to optimize anticoagulation strategy in nonvalvular atrial fibrillation. | Predicting the risk of thrombosis and bleeding in patients with nonvalvular atrial fibrillation (NVAF) who undergo catheter ablation (CA) | RF + XGBoost + GBT + LightGBM + Naïve Bayes (NB) + LR + NN + Deep neural network (DNN) | 844 | This study aimed to develop machine learning (ML) models to predict thrombosis and bleeding risks in nonvalvular atrial fibrillation (NVAF) patients undergoing catheter ablation (CA). Using data from 1055 patients, eight ML algorithms were compared, with XGBoost and Random Forest (RF) showing the best performance. Feature importance and SHAP analysis identified key predictors: with age, the duration of heparin, total bile acids (TBA), ALT, blood glucose, the level of BNP, fibrinogen, and low-density lipoprotein cholesterol (LDL_C). for thrombosis; and OAC category, dosage and duration, PLT, BNP, blood lipid, and albumin level for bleeding. RF-T and Xw-B models, using 25 and 27 features respectively, achieved good predictive accuracy (AUCs of 0.799 and 0.890).  ML models can be valuable tools for risk stratification and personalized anticoagulation strategies. However, the single-center nature and exclusion of some potentially important variables (like inflammatory markers) are limitations |
| 24. | Li 2024 | Predicting inferior vena cava filter complications using machine learning. | Predict 1-year IVC filter complications using preoperative data | Extreme Gradient Boosting (XGBoost) + random forest + Naïve Bayes classifier + radial basis function support vector machine + multilayer perceptron artificial neural network + and logistic regression | 10,133 | This study developed machine-learning models to predict 1-year complications after inferior vena cava (IVC) filter placement using preoperative data. Analyzing 14,476 patients, the best model (Extreme Gradient Boosting) achieved an AUROC of 0.93, outperforming logistic regression (AUROC 0.63). Key predictors included thrombophilia, prior VTE, antiphospholipid antibodies, and temporary filter placement. The model remained robust across subgroups and could aid clinical decision-making, patient counseling, and follow-up to reduce filter-related complications. |
| 25. | Fard 2024 | Machine learning analysis of bleeding status in venous thromboembolism patients. | Predicting bleeding risk for patients with venous thromboembolism (VTE) who are on extended anticoagulation therapy. | Unsupervised  PCA + kernel PCA + t-distributed stochastic neighboring embedding + isometric mapping + K-means clustering + agglomerative clustering  Supervised  Logistic regression + linear discriminant analysis + quadratic discriminant analysis (QDA) + Gaussian Naïve Bayes + SVM + adaptive boosting (AdaBoost) + gradient boosting | Not specified.  (Total cohort of 2,542 pa | A study using ML models to predict bleeding risk in VTE on anticoagulation using data from 2,542 VTE patients, 4.6% (118 individuals) of which had major bleeding found the 8 supervised ML models performed similarly to 6 traditional clinical risk models, with the best ML model only having an AUROC of ~67%. Unsupervised ML models however, were able to identify 2 clusters in patients who developed bleeding, one cluster consisting of females who had isolated DVT and experienced postthrombotic syndrome, another cluster which consisted of males who had isolated PE, wild-type VKORC1639, and heterozygous CYP4F2 mutation |
| 26. | Martin 2024 | Prediction model for major bleeding in anticoagulated patients with cancer-associated venous thromboembolism using machine learning and natural language processing | Assess the risk of major bleeding (MB) within 6 months in cancer patients receiving anticoagulant treatment for primary venous thromboembolism (VTE) | Logistic regression (LR) + decision tree (DT) + random forest (RF) | 1348 | This study developed a predictive model to assess the risk of major bleeding (MB) in cancer patients with venous thromboembolism (VTE) receiving anticoagulant treatment. Analyzing clinical data from 2.89 million patients, the model identified MB predictors such as hemoglobin, metastasis, age, platelets, leukocytes, and serum creatinine.  It used NLP to help to analyze the unstructured, free-text narratives in patients’ EHRs from nine Spanish hospitals. The ML models outperformed the traditional CAT-BLEED score but (AUROC = 0.53), but also only showed moderate predictive performance (AUROC ~0.61). It also found that Brain, esophageal, and lung cancers had the highest bleeding risks. |
| 27. | Contreras-Luján 2022 | Evaluation of Machine Learning Algorithms for Early Diagnosis of Deep Venous Thrombosis. | Diagnosis of deep vein thrombosis | DT + KNN + SVM + RF + Multi-layer perceptron neural network (MLP-NN) + extra trees (ET) | 8500 | This study explored the use of machine learning (ML) models for the efficient and reliable diagnosis of deep venous thrombosis (DVT). The input data in this study are the Wells criteria, the patient’s age, and the patient’s gender. The performance of different ML algorithms was evaluated, where KNN achieved the highest accuracy of 90.4% and speciﬁcity of 80.66% implemented on personal computer (PC) and Raspberry Pi 4 (RPi4).The accuracy of all trained models on PC and Raspberry Pi 4 is greater than 85%, while the area under the curve (AUC) values are between 0.81 and 0.86 |
| 28. | Nafee 2020 | Machine learning to predict venous thrombosis in acutely ill medical patients. | Predicting Venous Thromboembolism (VTE) | Ensemble learning combining 39 machine learning algorithms from 5 broad families  Families  Generalized additive models + elastic net + extreme gradient boosting + random forests + Bayesian logistic regression + simple classification tree | Not specified | This study compared the performance of machine learning (ML) models with the IMPROVE score for predicting venous thromboembolism (VTE) in acutely ill medical patients. The ML and rML algorithms outperformed the IMPROVE score in predicting VTE (c-statistic: 0.69, 0.68 and 0.59, respectively). Patients in the highest tertile had a 5-fold increase in odds of VTE compared to the lowest tertile. |
| 29. | Sheng 2023 | Development and validation of machine learning models for venous thromboembolism risk assessment at admission: a retrospective study. | Predicting venous thromboembolism (VTE) risk at admission | LR + RF + XGB | 2155 | This study aimed to develop and validate machine learning (ML) models to improve venous thromboembolism (VTE) risk assessment at hospital admission, comparing them to the traditional Caprini Risk Score (CRS). This was a retrospective study of 3078 individuals with Caprini variables collected within 24 hours of admission. The values of AUROC and AUPRC were 0.798 and 0.303 for LR, 0.804 and 0.360 for RF, and 0.796 and 0.352 for XGB, respectively, which outperformed CRS significantly (0.714 and 0.180, P < 0.001). When prediction scores were stratified into three risk levels for application, RF could obtain more reasonable results than CRS, including smaller false positive alerts and larger lower-risk proportions. |
| 30. | Wang 2021 | Prediction and Diagnosis of Venous Thromboembolism Using Artificial Intelligence Approaches: A Systematic Review and Meta-Analysis | Evaluate the performance of artificial intelligence (AI) in the diagnosis and prediction of venous thromboembolism (VTE) | ANN + Bayesian network + NLP + SVM | 33704 | This meta-analysis evaluated the performance of artificial intelligence (AI) in diagnosing and predicting venous thromboembolism (VTE). Out of 741 studies, 12 met the inclusion criteria. Among them, 5 studies included a training set and test set, and 7 studies included only a training set. In the training set, the pooled sensitivity was 0.87 (95% CI 0.79-0.92), the pooled specificity was 0.95 (95% CI 0.89-0.97), and the area under the summary receiver operating characteristic (SROC) curve was 0.97 (95% CI 0.95-0.98). In the test set, the pooled sensitivity was 0.87 (95% CI 0.74-0.93), the pooled specificity was 0.96 (95% CI 0.79-0.99), and the area under the SROC curve was 0.98 (95% CI 0.97-0.99). The combined results remained significant in the subgroup analyzes, which included venous thrombosis type, AI type, model type (diagnosis/prediction), and whether the period was perioperative. |
| 31. | Lu 2022 | Performance of multilabel machine learning models and risk stratification schemas for predicting stroke and bleeding risk in patients with non-valvular atrial fibrillation. | Prediction of outcomes and enhance decision-making for anticoagulant therapy in AF patients. | Classifier chains + SVM + GBM + MLNN | 7253 | This study compared multilabel machine learning (ML) models with traditional risk scores (CHA₂DS₂-VASc, HAS-BLED) for predicting stroke, major bleeding, and death in 9,670 non-valvular atrial fibrillation (AF) patients over 1 year.  Multilabel gradient boosting classifier chain provided the best AUCs for stroke (0.685 95% CI 0.676, 0.694), major bleeding (0.709 95% CI 0.703, 0.716) and death (0.765 95% CI 0.763, 0.768) compared to multi-layer neural networks and classifier chain using support vector machine. It provided modest performance improvement for stroke compared to AUC of CHA2DS2-VASc (0.652, NRI = 3.2%, p-value = 0.1), but significantly improved major bleeding prediction compared to AUC of HAS-BLED (0.522, NRI = 22.8%, p-value < 0.05). It also achieved greater discriminant power for death compared with AUC of CHA2DS2-VASc (0.606, p-value < 0.05). ML models identified additional risk features such as hemoglobin level, renal function. |
| 32. | Yang 2022 | An interpretable DIC risk prediction model based on convolutional neural networks with time series data. | Predict the risk of DIC in ICU patients. | XGBoost + LSTM + CNN | 4642 | This study developed an interpretable deep learning model to predict early risk of disseminated intravascular coagulation (DIC) in ICU patients. The model had good performance (AUC: 0.986, Accuracy: 95.7%, and F1:0.935). Gradient-weighted Class Activation Mapping (Grad-CAM) was used to explain how predictive models identified patients with DIC. |
| 33. | Fei 2017 | Artificial neural networks predict the incidence of portosplenomesenteric venous thrombosis in patients with acute pancreatitis | Predict the incidence of portosplenomesenteric venous thrombosis in patients with acute pancreatitis | ANN + LR | 48 | This study developed and validated an artificial neural network (ANN) model to predict portosplenomesenteric venous thrombosis (PSMVT) in acute pancreatitis (AP) patients and compared it with logistic regression. When the ANNs model was applied to the validation set, it revealed a sensitivity of 80%, specificity of 85.7%, a positive predictive value of 77.6% and negative predictive value of 90.7%. The accuracy was 83.3%. Differences could be found between ANNs modeling and logistic regression modeling in these parameters (10.0% [95% CI, -14.3 to 34.3%], 14.3% [95% CI, -8.6 to 37.2%], 15.7% [95% CI, -9.9 to 41.3%], 11.8% [95% CI, -8.2 to 31.8%], 22.6% [95% CI, -1.9 to 47.1%], respectively). When ANNs modeling was used to identify PSMVT, the area under receiver operating characteristic curve was 0.849 (95% CI, 0.807-0.901), which demonstrated better overall properties than logistic regression modeling (AUC = 0.716) (95% CI, 0.679-0.761). |
| 34. | Lu 2024 | Predicting multifaceted risks using machine learning in Atrial Fibrillation: Insights from GLORIA-AF study. | Predicting the risks of ischaemic stroke, major bleeding, and death within one year in patients with atrial fibrillation | Multi-label gradient boosting decision tree (ML-GBDT) + | 17,959 | This study evaluated the multi-label gradient boosting decision tree (ML-GBDT) model for predicting death, ischemic stroke, and major bleeding in 25,656 atrial fibrillation (AF) patients. The model achieved an optimized area under the curve in predicting death (0.785, 95% CI: 0.757-0.813) compared with the Charlson Comorbidity Index (0.747, P = 0.007), ischaemic stroke (0.691, 0.626-0.756) compared with CHA2DS2-VASc (0.613, P = 0.028), and major bleeding (0.698, 0.651-0.745) as opposed to HAS-BLED (0.607, P = 0.002), with improvement in net reclassification index (10.0, 12.5, and 23.6%, respectively). |
| 35. | Mora 2021 | Machine Learning to Predict Outcomes in Patients with Acute Pulmonary Embolism Who Prematurely Discontinued Anticoagulant Therapy. | Identifying patients at risk for the composite outcome of fatal PE or recurrent VTE within 30 days after premature discontinuation of anticoagulation therapy. | Decision tree + KNN + SVM + Ensemble + NN + logistic regression |  | This study used machine learning (ML) models to predict fatal pulmonary embolism (PE) or recurrent venous thromboembolism (VTE) within 30 days after premature anticoagulation discontinuation (<90 days) in 34,447 PE patients from the RIETE registry. ML-NN was the best method for identification of patients experiencing the composite endpoint, predicting the composite outcome with an area under receiver operating characteristic (ROC) curve of 0.96 (95% confidence interval [CI]: 0.95-0.98), using either 70 or 23 variables captured before discontinuation. The discrimination of logistic regression was inferior (area under ROC curve, 0.76 [95% CI: 0.70-0.81]). |
| 36. | El-Bouri 2023 | Predicting acute and long-term mortality in a cohort of pulmonary embolism patients using machine learning. | Predicting mortality in PE patients over different timeframes (30, 90, and 365 days) | Random forest + XGBoost + logistic regression | 1528 | This study developed a machine learning (ML) risk score to predict 30-day, 90-day, and 365-day mortality in pulmonary embolism (PE) patients using data from the BBC-VTE registry (2,183 patients). The machine learning risk score predicted 30-day mortality with AUC 0.71 [95% CI: 0.63 - 0.78] compared to the sPESI AUC of 0.65 [95% CI: 0.57 - 0.73] and PESI AUC of 0.64 [95% CI: 0.56 - 0.72]. 90-day mortality and 365-day mortality were predicted with an AUC of 0.74 and 0.73 respectively. Despite being significant, however, the difference in AUC, specificity and sensitivity is not large (in the order of 2 – 12 %). |
| 37. | Peng 2024 | Computed tomography-based multi-organ radiomics nomogram model for predicting the risk of esophagogastric variceal bleeding in cirrhosis | Predict risk of first-instance secondary esophageal variceal bleeding in patients with cirrhosis | LASSO regression analysis | 145 | The study aimed to develop a computed tomography (CT)-based multi-organ radiomics nomogram model for predicting esophagogastric variceal bleeding (EVB) in cirrhosis. Unlike traditional single-organ image analysis, this model incorporates radiomic features from the liver, spleen, and lower esophagus-gastric fundus region to improve non-invasive risk assessment. 208 cirrhotic patients were retrospectively analyzed and divided into training (n=145) and validation (n=63) cohorts.  Clinical risk factors were also analyzed, with ascites, portal vein thrombosis (PVT), and plasma prothrombin time (PT) identified as key predictors.  The RC model had the highest predictive accuracy for EVB risk (AUC = 0.951 in training, 0.930 in validation), outperforming models using only single-organ radiomic data or clinical data alone. |
| 38. | Chen 2023 | Application of artificial neural network in daily prediction of bleeding in ICU patients treated with anti-thrombotic therapy. | Predicting daily bleeding risk in ICU patients receiving anti-thrombotic therapy | RNN | 800 | A prediction model was built using RNNs to predict daily bleeding risk in ICU patients on anti-thrombotic drugs. The model's performance was compared to predictions made by junior and senior clinicians. 938 patients were incorporated into the study, and at the meantime, 1000 non-bleeding patients were randomly extracted. |

#### **Accelerating Drug Development**

| No. | Author | Title | Objective | Artificial Intelligence Model/Algorithm | Training set size | Summary |
| --- | --- | --- | --- | --- | --- | --- |
| 1. | Cosín-Sales 2024 | Safety and Effectiveness of Oral Anticoagulants in Atrial Fibrillation: Real-World Insights Using Natural Language Processing and Machine Learning | Compare the effectiveness and safety of vitamin K antagonists (VKAs) versus direct oral anticoagulants (DOACs) in patients with atrial fibrillation (AF) | NLP + machine learning | NA | This retrospective study analyzed 44,292 atrial fibrillation (AF) patients from 15 Spanish hospitals (2014–2020) using AI techniques to compare vitamin K antagonists (VKAs) and direct oral anticoagulants (DOACs). DOAC users had lower risks of thrombotic events (HR 0.81), minor bleeding (HR 0.89), and mortality (HR 0.80) than VKA users. The study demonstrates that DOACs offer a safer and more effective alternative to VKAs in real-world AF patients. |
| 2. | Rovenchak 2024 | Machine learning-assisted search for novel coagulants: When machine learning can be efficient even if data availability is low | Suggest new inhibitor candidates by developing a deep learning-based approach for building an effective representation of chemical space | SVM + RF + LightGBM + XGBoost | Not specified | This research paper investigates the application of machine learning (ML) techniques for suggesting novel compounds, specifically coagulants with a specific case of limited data availability.  The autoencoder successfully mapped a chemical space of small organic molecules.  Clustering analysis in the embedding space revealed distinct clusters for Thrombin and Protein C inhibitors.  Both interpolation and hypersphere search methods generated novel candidate molecules. The researchers were able to create a working model that can be used to create new coagulants. |
| 3. | Faquetti 2022 | Identification of novel off targets of baricitinib and tofacitinib by machine learning with a focus on thrombosis and viral infection | Identify potential off-target interactions of the JAK inhibitors baricitinib and tofacitinib that could explain the increased risks of thrombosis and viral infection/reactivation associated with their use | Neural network | NA | The study investigates potential off-target effects of the Janus kinase (JAK) inhibitors, baricitinib and tofacitinib, which have been linked to increased risks of thrombosis and viral infections/reactivation.  Two Machine learning approaches (TIGER and SPiDER) based on ligand similarity were used to predict potential off-targets.  In vitro assays validated the interactions for targets related to thrombosis and viral infections.  Computational ligand docking then helped predict potential binding mechanisms.  Four inhibitory interactions were confirmed:  Baricitinib: Inhibited PDE10A (linked to pulmonary vascular remodeling) and PKN2.  Tofacitinib: Inhibited TRPM6 (related to magnesium homeostasis) and PKN2.  These interactions did not explain the increased risk of thrombosis or viral infections/reactivation.  Instead, they suggested potential benefits, such as:  PDE10A inhibition may help in pulmonary arterial hypertension.  PKN2 inhibition might modulate HCV viral response.  TRPM6 inhibition could be linked to hypomagnesemia.  The drugs were shown to inhibit two targets related to thrombosis—PDE10A and TRPM6. PDE10A, which was recently validated as a novel target to treat pulmonary arterial hypertension (PAH) due to its central role in progressive pulmonary vascular remodeling. Clinically, PDE10A inhibition is expected to decrease the risk for thrombosis, particularly in patients with PAH. Thus, the expected positive clinical impact of PDE10A inhibition on the risk of thrombosis is not in line with a potential link to an elevated thrombosis risk. Rather, baricitinib might improve progressive pulmonary vascular remodeling.  Cumulatively, the active targets in this study suggest that JAK inhibitors may have a beneficial effect on cardiovascular risk, and therefore do not support a hypothesis that the risk of thrombosis is related to an off-target drug effect (in the framework of the macromolecular targets investigated in this study). Nevertheless, we note  that recent US-based cohort studies that have identified no difference in thrombosis risk between tofacitinib and TNF-inhibitors, thereby suggesting that much of the observed risk seen in pharmacovigilance studies may be due to underlying risk factors rather than a drug effect. |
| 4. | Masand 2024 | GA-XGBoost, an explainable AI technique, for analysis of thrombin inhibitory activity of diverse pool of molecules and supported by X-ray. | Identifying key structural features that govern thrombin inhibitory activity. | XGBoost + random forest + Naïve Bayes classifier + radial basis function SVM + MLP-NN + logistic regression | 10,133 | This study focuses on using a combination of extreme gradient boosting (XGBoost), Shapley values, and genetic algorithms to analyze the thrombin inhibitory activity of a large set of molecules (2803). The analysis revealed that aromatic carbon and ring/non-ring nitrogen, in combination with other structural features, play a crucial role in determining the thrombin inhibitory profile. |
| 5. | Xie 2017 | Identification of potential drug targets based on a computational biology algorithm for venous thromboembolism. | Identifying recurrent risk modules (RRMs) associated with venous thromboembolism (VTE). | SVM | Not specified | This study investigates recurrent venous thromboembolism (VTE) using a computational biology approach to identify potential drug targets.  Seven Recurrent Risk Modules (RRMs) were identified, mainly involved in coagulation cascades, blood circulation, apoptosis, and signal transduction. RRM M5 had the strongest link to VTE recurrence, containing genes associated with coagulation (F2, F5, F7, F10, etc.). Nine approved drug targets were identified within these RRMs, particularly in M5, suggesting potential targets for drug repurposing.  Warfarin and other anticoagulants (e.g., apixaban, dabigatran) target these RRMs, highlighting their importance in VTE treatment. |
| 6. | Datta 2021 | ‘Black Box’ to ‘Conversational’ Machine Learning: Ondansetron Reduces Risk of Hospital-Acquired Venous Thromboembolism‘Black Box’ to ‘Conversational’ Machine Learning: Ondansetron Reduces Risk of Hospital-Acquired Venous Thromboembolism, | Identify factors affecting the risk of hospital-acquired venous thromboembolism (HA-VTE) | Logistic regression | 397,064 | This study explores how machine learning (ML) and electronic healthcare records (EHRs) can identify drugs that reduce the risk of hospital-acquired venous thromboembolism (HA-VTE).  It used a 'conversational approach' where the most important variables in the model were identified, then domain experts  reviewed these variables and identified those that are likely  to be surrogate markers or confounding variables, with the models being retrained until no more such variables were identified. Logistic regression was employed as the ML technique.  This study used records of 397,064 hospitalizations from the BJC healthcare network at St. Louis MO, which includes 2181 (0.54%) VTE positives.  It identified ondansetron, an anti-nausea drug, reduces the risk of HA-VTE (relative risk reduction [RRR] of 11%)—comparable to aspirin (15.5%).  A temporal control using D-dimer tests ensured that observed effects were not biased by prior VTE diagnoses. After D-dimer masked analysis, Aspirin and ondansetron have a relative risk reduction of around 16.2% and 8.9% respectively  Ondansetron’s effect was also dose-dependent, strengthening its potential as a repurposed therapy.  A potential biological mechanism is that ondansetron appears to inhibit platelet aggregation through pathways involving MAPK and IP3 signaling |
| 7. | Kamola 2024 | Qualitative classification of thrombus images as a way to improve quantitative analysis of thrombus formation in flow chamber assays. | Improve the quantification of thrombus formation in flow chamber assays by incorporating machine learning-based classification of thrombi based on morphological features | Not specified | Not specified | The study uses **machine learning (Ilastik)** to improve **thrombus classification in flow chamber assays**. By distinguishing **compacted vs. non-compacted thrombi**, ML enhances **quantification accuracy** and **better detects antiplatelet drug effects**. This automated approach improves **thrombosis research and drug evaluation**. |
| 8. | Ramírez-Galicia 2012 | Exploring QSARs for inhibitory effect of a set of heterocyclic thrombin inhibitors by multilinear regression refined by artificial neural network and molecular docking simulations. | Predicting thrombin inhibition by non-peptide heterocyclic compounds | Multiple linear regression (MLR) + ANN | 50 | The study explores the Quantitative Structure-Activity Relationship (QSAR) of heterocyclic thrombin inhibitors using multiple linear regression (MLR) and artificial neural networks (ANN), combined with molecular docking simulations. 50 non-peptide heterocyclic thrombin inhibitors were analysed. Ligand binding sites were found at thrombin sites S1 and D |
| 9. | Huang 2019 | Computer-aid drug design, synthesis, and anticoagulant activity evaluation of novel dabigatran derivatives as thrombin inhibitors. | Design, synthesize, and evaluate novel dabigatran derivatives with anticoagulant potential using computer-aided drug design (CADD) techniques | QSAR (CoMFA, CoMSIA) | Not specified | This research paper focuses on the development of novel thrombin inhibitors. The study employs a combination of computational and experimental methods. Computational methods used included 3D-QSAR models (CoMFA and CoMSIA) to understand the structural features that contribute to thrombin inhibition.  Employed molecular docking to predict how the designed compounds would bind to the thrombin enzyme.  Performed molecular dynamics simulations to assess the stability of the compound-enzyme interactions. Used ADME prediction to determine drug like qualities.  They also synthesized 11 new compounds based on the computational design. The results of the anticoagulant activity showed that the bioactivity of all the designed compounds were comparable to that of the reference dabigatran. In particular, 12a, 12c and 12g showed better anticoagulant activities with IC50 values of  11.19 ± 1.70, 10.94 ± 1.85 and 11.49 ± 2.57 nM, respectively, which were equivalent to the dabigatran. |

####

#### **Others**

| No. | Author | Title | Objective | Artificial Intelligence Model/Algorithm | Training set size | Summary |
| --- | --- | --- | --- | --- | --- | --- |
| 1. | Nguyen 2021 | Comparison of multivariate linear regression and a machine learning algorithm developed for prediction of precision warfarin dosing in a Korean population. | Develop and compare personalized warfarin dosing models to accurately predict stable warfarin doses | LR + GBM | 585 | This study compared multiple linear regression (LR) and gradient boosting machine (GBM) for personalized warfarin dosing in a Korean population (650 patients). LR and GBM models were comparable in terms of accuracy of ideal dose (75.38% and 73.85%), correlation (0.77 and 0.73), mean absolute error (0.58 mg/day and 0.64 mg/day), and root mean square error (0.82 mg/day and 0.9 mg/day), respectively. LR may be the appropriate model due to its simplicity and interpretability. |
| 2. | Hasegawa 2020 | Comparative Analysis of Three Machine-Learning Techniques and Conventional Techniques for Predicting Sepsis-Induced Coagulopathy Progression. | Evaluate predictive models for sepsis-induced coagulopathy progression | LR + RF + SVM + NN | 712 | This study focused on developing and evaluating predictive models for sepsis-induced coagulopathy progression, using machine learning (ML) techniques and comparing their performance with conventional methods. A post-hoc subgroup analysis was conducted based on the Japan Septic Disseminated Intravascular Coagulation retrospective study. Regarding DIC progression, predictive accuracy of the multiple linear regression, RF, SVM, and NN models was 63.7%, 67.0%, 64.4%, and 59.8%, respectively. The difference between predicted ΔDIC and real ΔDIC was 2.05, 1.54, 2.24, and 1.77 for the multiple linear regression, RF, SVM, and NN models, respectively. |
| 3.. | McInnes 2019 | Predicting venous thromboembolism risk from exomes in the Critical Assessment of Genome Interpretation (CAGI) challenges | Assess the feasibility and effectiveness of predicting venous thromboembolism (VTE) status from exome sequencing data in African American individuals | PCA + k-means clustering + deep learning + logistic regression | NA | Participants were provided with 103 unlabeled exomes from patients treated with warfarin for non-VTE causes or VTE and asked to predict which disease each subject had been treated for. Given the lack of training data, many participants opted to use unsupervised machine learning methods, clustering the exomes by variation in genes known to be associated with VTE. The best performing method using only VTE related genes achieved an area under the ROC curve of 0.65. Here, we discuss the range of methods used in the prediction of VTE from sequence data and explore some of the difficulties of conducting a challenge with known confounders |
